# Genomic Diversity of Vaginal *Lactobacillus crispatus* Prophages from South African Women

**DOI:** 10.3390/v18050519

**Published:** 2026-04-30

**Authors:** Adijat Ozohu Jimoh, Anika Chicken, Brandon Maust, Colin Feng, Seth Rakoff-Nahoum, Jo-Ann S. Passmore, Brian R. Kullin, Simona Kraberger, Fatima Aysha Hussain, Heather B. Jaspan, Arvind Varsani, Anna-Ursula Happel

**Affiliations:** 1 Division of Immunology, Department of Pathology, University of Cape Town, Cape Town 7925, South Africa; jmhadi001@myuct.ac.za (A.O.J.); heather.jaspan@uct.ac.za (H.B.J.); 2 Institute of Infectious Disease and Molecular Medicine, University of Cape Town, Cape Town 7925, South Africa; 3 Genetics, Genomics, and Bioinformatics Department, National Biotechnology Research and Development Agency, Abuja 900107, Nigeria; 4 Division of Medical Virology, Department of Pathology, University of Cape Town, Cape Town 7925, South Africa; anikachicken@sun.ac.za (A.C.); jo-ann.passmore@uct.ac.za (J.-A.S.P.); brian.kullin@uct.ac.za (B.R.K.); 5 Division of Molecular Biology and Human Genetics, Faculty of Medicine and Health Sciences, Stellenbosch University, Tygerberg, Cape Town 7925, South Africa; 6 Center for Global Infectious Disease Research, Seattle Children’s Research Institute, Seattle, WA 98109, USA; brandon.maust@seattlechildrens.org (B.M.); colin.feng@seattlechildrens.org (C.F.); 7 Department of Pediatrics, University of Washington School of Medicine, Seattle, WA 98195, USA; 8 Division of Infectious Diseases, Boston Children’s Hospital and Harvard Medical School, Boston, MA 02115, USA; seth.rakoff-nahoum@childrens.harvard.edu; 9 National Health Laboratory Services, Cape Town 7925, South Africa; 10 The Biodesign Center for Fundamental and Applied Microbiomics, Center for Evolution and Medicine and School of Life Sciences, Arizona State University, Tempe, AZ 85281, USA; simona.kraberger@asu.edu (S.K.); avarsani@gmail.com (A.V.); 11 Department of Biology, Tufts University, Medford, MA 02155, USA; fatima.hussain@tufts.edu; 12 Department of Global Health, University of Washington, Seattle, WA 3980, USA; 13 Structural Biology Research Unit, Department of Integrative Biomedical Sciences, University of Cape Town, Cape Town 7925, South Africa

**Keywords:** *Lactobacillus crispatus*, bacteriophage genomics, prophage induction, vaginal microbiota

## Abstract

*Lactobacillus crispatus* is widely associated with optimal sexual and reproductive health outcomes. While *L. crispatus* genomes commonly harbor prophages, little is known about their genomic diversity and potential inducibility by clinically relevant compounds. We induced and characterized four bacteriophages from four *L. crispatus* strains isolated from vaginal secretions of South African adolescents. Sequenced viral DNA from induced phages was assembled, and their respective genomes were annotated and compared to bacteriophage reference genomes. All the phage genomes range in size from 42.9 to 48.3 kbp. Of the four phages, UC101 and UC164 shared <90% pairwise intergenomic similarity to reference phages, suggesting that they represent new species. To explore factors potentially associated with prophage activation, *L. crispatus* strains were exposed to physiological concentrations of copper ions and tenofovir, selected based on their common use by women in Africa and reported associations with altered vaginal bacterial community composition. The presence of phage-like particles following exposure to copper ions (2.0 × 10^−6^ M–3.0 × 10^−6^ M) and tenofovir (500 ng/mL) was observed by transmission electron microscopy, suggesting possible prophage activation under these conditions. This study provides new insights into the genomic diversity of inducible *L. crispatus* phages and presents hypothesis-generating evidence regarding their potential inducibility using copper ions and tenofovir.

## 1. Introduction

An optimal vaginal microbiota is associated with protection against sexually transmitted infections (STIs) and adverse reproductive health outcomes, such as preterm birth [1] and early-onset neonatal sepsis [2]. Vaginal *Lactobacillus crispatus* is thought to be a key player facilitating these health benefits [3,4,5]. Displacement of *L. crispatus* by diverse facultative anaerobes can result in bacterial vaginosis (BV), a globally prevalent condition in women of reproductive age, with recently reported prevalences between 45% and 50% in cohorts of South African women [6,7]. BV has been linked to increased risk of STIs, pelvic inflammatory diseases, and adverse birth outcomes [8,9,10,11,12].

Besides bacteria, the female genital tract (FGT) also harbors viruses that contribute to its microbial ecology, including temperate bacteriophages [13]. When integrated into bacterial genomes as prophages, they can provide advantages to their bacterial host by improving their virulence, fitness, and environmental adaptation [14,15,16]. Several studies have identified prophages in vaginal bacterial genomes [17,18,19,20]. A South African metagenomic study identified five distinct vaginal *L. crispatus* prophage clusters, possibly indicating intra- and inter-host diversity [18]. Furthermore, pangenome analyses of 105 *L. crispatus* genomes from diverse niches demonstrated that human-derived isolates were more likely to harbor intact complete prophages than non-human isolates, which could suggest that prophages are an important component of the genome evolution of *L. crispatus* in the vagina [21]. Notably, studies have demonstrated the inducibility of bacteriophages from *L. crispatus* and *L. jensenii* strains isolated from vaginal samples of South African women [22]. However, the genomic diversity of vaginally derived *L. crispatus* prophages from African women remains under-characterized, and most experimental studies on *Lactobacillus*-infecting bacteriophages have focused on dairy strains [23,24,25]. This leaves knowledge gaps related to the genetic diversity of vaginal *L. crispatus* prophages and their genomic features. Here, we report the whole-genome characterization of prophages from vaginal *L. crispatus* strains isolated from South African women, thereby contributing phage genomes to currently underrepresented vaginal virome databases.

Understanding how these prophages may respond to physiologically relevant chemical exposures is important in the context of women’s reproductive health. Women in sub-Saharan Africa are at risk for both unintended pregnancies and human immunodeficiency virus (HIV). The copper intrauterine device (Cu-IUD), one of the few non-hormonal contraceptives available in low- and middle-income countries [26,27], has been associated with altered vaginal microbiota composition and genital inflammation [28,29,30], although the underlying mechanisms are unknown. In parallel, the use of tenofovir (TFV)-based HIV pre-exposure prophylaxis (PrEP) is expanding [31], and interactions between vaginal microbial communities and TFV efficacy have been reported [32]. Chemical exposures, including compounds found in cigarette smoke, have been shown to induce bacteriophages from vaginal *Lactobacillus* strains *in vitro* [33], raising the possibility that other commonly encountered compounds, such as copper ions and TFV, may influence prophage induction in vaginal *L. crispatus* strains. This study explores this possibility in a hypothesis-generating manner.

## 2. Materials and Methods

Study cohort: HIV-seronegative sexually active South African female adolescents aged 15–19 years were recruited into the parent study (UChoose; clinicaltrials.gov registration number: NCT02404038) [34]. Ethics approval was obtained from the University of Cape Town’s Human Research Ethics Committee for the parent study (HREC 801/2014) and for this sub-study (HREC 657/2022). Vaginal swabs were collected from consenting study participants by trained research nurses, including vaginal lateral swabs for bacterial culture that were stored in 1× phosphate-buffered saline (PBS) supplemented with 60% glycerol stored at −80 °C.

Isolation of *Lactobacillus* strains: Vaginal bacteria were isolated by streaking a 10-fold dilution series of the vaginal swab solution onto de Man, Rogosa and Sharpe (MRS) agar (Merck Millipore, Johannesburg, South Africa) plates supplemented with 0.05 g/L L-cysteine hydrochloride monohydrate (Sigma-Aldrich, Tokyo, Japan), followed by incubation at 37 °C in an anaerobic chamber (Baker Ruskinn Concept, Bridgend, UK) in an atmosphere consisting of 5% hydrogen, 10% carbon dioxide and 85% nitrogen with a relative humidity of 60%. Single colonies were picked based on colony morphology, and pure cultures were obtained by repeated sub-culturing. For long-term storage, these strains were then stored in 18% (*v*/*v*) glycerol at −80 °C [35]. Species identity of pure strains was confirmed by full-length 16S rRNA gene Sanger sequencing. A previously described colony PCR protocol was used with the universal 16S rRNA gene primers F27 and R5 [36]. Products were Sanger sequenced (Inqaba Biotec, Pretoria, South Africa) using the 907R primer [37] and the resulting sequences classified by comparing to the NCBI 16S rRNA gene database using the BLAST algorithm v2.16.0+ [38]. Frozen stocks of stored *L. crispatus* strains were sub-cultured onto MRS agar (1.5%) plates supplemented with 0.05 g/L L-cysteine hydrochloride monohydrate. The plates were incubated in an anaerobic chamber (Baker Ruskinn, Concept, UK) with atmospheric conditions of 10% carbon dioxide, 85% nitrogen, 5% hydrogen, and 60% relative humidity for 24–48 h.

Whole-genome sequencing of *L. crispatus* strains: Bacterial DNA was extracted from single colonies of pure *L. crispatus* strains using the Purelink Genomic DNA mini kit (Invitrogen, Thermo Fisher Scientific, Waltham, MA, USA), as per manufacturer’s instructions. Libraries were constructed using the Illumina Nextera Kit (Illumina, San Diego, CA, USA), and DNA was sequenced on an Illumina NovaSeq 6000 platform, generating 2 × 250 bp pair-end reads. Read quality was assessed with FastQC v0.12.1 [39] and low-quality reads trimmed with fastp v0.23.4 [40]. Trimmed reads were assembled *de novo* into draft bacterial genomes using SPAdes v4.0.0 [41], and assembly metrics were assessed using QUAST v5.2.0 [42]. Assembled genome contigs were annotated using Prokka v1.14.6 [43].

*In silico* prophage identification in *L. crispatus* genomes: VirSorter2 (v2.2.4) [44] using the following parameters: “--include-groups dsDNAphage, ssDNA-min-length 5000-min-score 0.5” was used to identify prophages in the bacterial genomes. This was followed by checkV analysis (v1.0.3) [45] to assess viral genome completeness and PHASTER (https://bio.tools/phaster) (accessed on 24 August 2024) [46] to identify putative integration sites.

*In vitro* prophage induction using mitomycin C: Four vaginal *L. crispatus* strains (UC1640140, UC0930205, UC1190127, and UC1010110) predicted with high confidence to harbor prophages were selected for experimental prophage induction with mitomycin C (Thermo Fisher Scientific, Waltham, MA, USA). Each strain was sub-cultured onto MRS agar plates as described above. Single colonies from each strain were used to inoculate 5 mL of pre-reduced (overnight incubation under anaerobic conditions to deoxygenate before use) MRS broth supplemented with 0.05 g/L L-cysteine hydrochloride monohydrate (Sigma-Aldrich, Tokyo, Japan) and incubated for 12–18 h at 37 °C under anaerobic conditions as described above. Bacterial cultures were diluted to an OD_600_ of 0.1 in 50 mL MRS broth supplemented with 10 mM CaCl_2_, 0.05 g/L L-cysteine hydrochloride monohydrate, and incubated for 3 h at 37 °C under anaerobic conditions until reaching early exponential phase (OD_600_~0.3). At this point, mitomycin C at a final concentration of 0.5 µg/mL was added, as previously described [18]. Growth was monitored in bacterial cultures with and without mitomycin C over 18–24 h. Bacterial cultures were centrifuged at 4000× *g* for 12 min at 4 °C to remove bacterial cellular debris, and the supernatant was filtered using 0.45-µm cellulose-acetate-based syringe filters (Lasec, Cape Town, South Africa).

Transmission electron microscopy (TEM): For TEM, 1.5 mL of filtrate was centrifuged at 21,000× *g* for 1 h at 4 °C (Sigma 3-16KL refrigerated benchtop centrifuge, Osterode am Harz, Germany), and pellets were resuspended in 100 µL of SM buffer (Thermo Scientific Alfa Aesar, Ward Hill, MA, USA). Centrifugation was repeated, after which the pellet was resuspended in 50 µL of SM buffer and stored at 4 °C. Carbon-coated copper grids (Agar Scientific, Stansted, UK) were rendered hydrophilic using an EMS100 Glow Discharge Unit (Electron Microscopy Sciences, Hatfield, PA, USA). Five µl of sample was placed on each grid and incubated for 10 min at room temperature. Each grid was washed with 20 µL of distilled water and then stained with 10 µL of 2% uranyl acetate (SPI Supplies, West Chester, PA, USA) for 3 min. Imaging was performed using an FEI T20 (Thermo Fisher (FEI), Eindhoven, Netherlands) at 200 kV using the Gatan US 1000 CCD camera (Ametek Inc., Pleasanton, CA, USA) at the Electron Microscope Unit, University of Cape Town. Sizes of bacteriophage capsids were measured using ImageJ software (v1.54).

Whole-genome sequencing of induced phages: Prior to DNA extraction, filtered phage-containing supernatants (~45 mL) were concentrated using 100 kDa Amicon Ultra-15 Centrifugal filters (Merck, Darmstadt, Germany) by centrifugation (3000× *g*, 5 min, 4 °C) based on an adapted protocol [47] with a reduced centrifugation speed. The phage concentrate was washed by adding 15 mL of filtered SM buffer to the upper reservoir and centrifuged again at 3000× *g* for 5 min. This wash step was repeated until the volume of the phage concentrate was ~2 mL and appeared clear. After transferring the phage concentrate to a new tube, the upper chamber was rinsed with 500 µL of SM buffer to recover residual phages adhering to the membrane surface. The rinsed volume was added to the phage concentrate and stored at 4 °C. Phage DNA was extracted using a published protocol [48] with modifications to the DNA resuspension volumes. Briefly, 1.5 µL each of DNase I and RNase A (Thermo Fisher Scientific, Waltham, MA, USA) was added to 600 µL phage concentrate to digest free DNA/RNA and incubated at 37 °C for 1 h. Further, 1.5 μL of Proteinase K (20 mg/mL) (Qiagen, Hilden, Germany), 30 μL of 10% SDS (Merck Darmstadt, Germany), and 24 μL of 0.5 M EDTA (pH 8.0) were added and incubated for 1 h at 60 °C, while vortexing at 20-min intervals, to digest proteins. Subsequently, an equal volume of phenol:chloroform (1:1) (Invitrogen, Thermo Fisher Scientific, Waltham, MA, USA) was added to the solution and inverted several times before centrifugation at 18,000× *g* for 5 min at room temperature. This step was repeated after transferring the supernatant to a new 2 mL tube. An equal volume of chloroform was added to the supernatant (1:1), inverted to mix well, centrifuged as above, before transferring the supernatant to a new 2 mL tube. DNA was precipitated by adding 0.1 volume of 3 M sodium acetate (pH 7.5) and 2.5 volumes of 100% ice-cold ethanol to the final aqueous phases from each aliquot. Sample tubes were inverted repeatedly, incubated overnight at −20 °C and then centrifuged for 20 min at 20,000× *g* at room temperature. Pelleted DNA was washed with freshly prepared 70% ethanol and centrifuged again as described above. This wash step was repeated once. The ethanol was removed without disturbing the pellet, and the tubes were left to air dry in a biosafety cabinet for 10 min. The DNA pellet was resuspended in 50 μL of TE buffer (pH 7.6), and the DNA concentration was quantified with a Qubit fluorometer using the Qubit dsDNA High Sensitivity Assay Kit (Thermo Fisher Scientific, Waltham, MA, USA). Illumina sequencing libraries were generated from the extracted phage DNA using the Illumina DNA library preparation (M) tagmentation kit (Illumina, San Diego, CA, USA) and sequenced on an Illumina NovaSeq X Plus sequencer (Illumina, San Diego, CA, USA) at Psomagen Inc. (Rockville, MD, USA). The 2 × 150 bp pair-end reads were trimmed using Trimmomatic v0.39 [49] and *de novo* assembled using MEGAHIT v1.2.9 [50]. The resulting viral contigs were first checked using Cenotetaker 2 [51] using the discovery mode. Phages UC101 and UC119 had insufficient read depth, therefore complete genomes of these two phages were reconstructed based on the integration and the att sites within the bacterial host genomes. Phages UC093 and UC164 had sufficient read coverage for a full genome assembly and no reconstruction was needed. The complete bacteriophage genomes with terminal redundancy were annotated using Pharokka v1.7.5 [52] with refinement using phold v0.2.0 (https://github.com/gbouras13/phold) (accessed on 8 April 2025).

Computational analysis of bacteriophage genomes: Bacteriophage integration sites were identified through sequence alignment with MAFFT v7.490 [53] of phage and bacterial host genomes in Geneious Prime v2025.0.3 (https://www.geneious.com) (accessed on 11 April 2025). Phage attachment sites in the bacterial host genomes were predicted by PHASTER [46]. To further characterize the phage genomes, phage sequences that shared >75% identity with >75% BLASTn genome coverage were downloaded from GenBank and were used to infer a proteomic phylogenetic tree using the ViPTree web server (https://www.genome.jp/viptree/) (accessed on 14 April 2025) [54]. The sequences within the two major clades of the ViPTree phylogeny were then used to determine intergenomic similarities using VIRIDIC [55]. Gene cluster comparisons of annotated protein sequences of induced phage genomes were performed using the Clinker software [56] to highlight conserved and variable genome regions between phages. To validate prophage predictions, induced phage genomes were aligned with predicted prophage regions from their respective host strains using MAFFT v7.490 [53].

*In vitro* prophage induction using clinically relevant agents: *L. crispatus* strains confirmed to harbour inducible prophages via mitomycin C induction and TEM were exposed to clinically relevant concentrations of copper and TFV. Copper sulfate (Medic+, Cape Town, South Africa) dilutions corresponding to final concentrations of copper ions at 1.5 × 10^−6^ M, 2.0 × 10^−6^ M, 2.5 × 10^−6^ M, and 3.0 × 10^−6^ M were tested [28,57,58]. TFV (Sigma-Aldrich, St. Louis, MO, USA) was tested at final concentrations of 500, 1000, 2000, 4000, and 8000 ng/mL [32,59,60,61]. To monitor bacterial growth kinetics during phage induction experiments with copper sulfate and TFV, *L. crispatus* strains were first grown in 10 mL pre-reduced MRS broth supplemented with 0.05 g/L L-cysteine hydrochloride monohydrate for 12–18 h under anaerobic conditions. Bacterial cultures were diluted to an OD_600_ of 0.1 in fresh MRS broth (supplemented with 10 mM CaCl_2_ and 0.05 g/L L-cysteine hydrochloride monohydrate) and dispensed in triplicate into 96-well plates for each test concentration. The 96-well plate was placed in the Stratus Kinetic Microplate Reader (Cerillo, Charlottesville, VA, USA), and bacterial cultures were grown under anaerobic conditions until reaching early exponential phase, at which point the experimental compounds were added in different concentrations. Media negative controls (culture media only), bacterial culture negative controls (bacteria only in the absence of the test inducers), and positive controls for phage induction (0.5 µg/mL mitomycin C) were included in all phage induction experiments. OD_600_ was recorded every 30 min over 24 h. Post-incubation, the OD_600_ readings were analyzed using the Cerillo Labrador Software, with statistical analyses and growth curve visualization done with RStudio (version 4.4.0; http://www.rstudio.com). Growth curves were used to document bacterial growth dynamics in the presence or absence of test inducers. Phage induction was verified by TEM, as described above, to confirm induced phage particles in supernatants from experimental culture treatments and their absence in untreated negative bacterial culture controls and media controls.

## 3. Results

### 3.1. In Vitro Prophage Inductions from Vaginal L. crispatus Strains Using Mitomycin C

We identified prophages in the genomes of *L. crispatus* strains isolated from vaginal secretions of South African women. As these bacterial host genomes were draft genomes, viral prophage regions spanned multiple contigs of host genomes. Thus, only *Lactobacillus* strains (UC0930205, UC1010110, UC1190127, and UC1640140) with predicted prophage regions classified as intact by PHASTER or of high or medium quality by CheckV were retained, and low-quality predictions were excluded. Three *L. crispatus* strains (UC0930205, UC1010110, and UC1190127) were predicted to each contain one intact prophage. The UC0930205 and UC1010110 strains were each predicted to harbor two additional incomplete prophages. *L. crispatus* strain UC1640140 was predicted to harbor one prophage of medium quality (completeness estimate: 83.7%), likely reflecting fragmentation of the prophage across bacterial contigs. These predicted prophages ranged from 23,956 to 42,894 bp in size, with a GC content range of 35.4–40% and genome completeness estimates of 64.66–100%. BLASTn comparisons of these predicted prophage sequences revealed 94.20–99.97% nucleotide identity to *Caudoviricetes* phages, all of which were identified in the same study from the vaginal fornix of a female participant [51] (Table 1).

**Figure 1:**
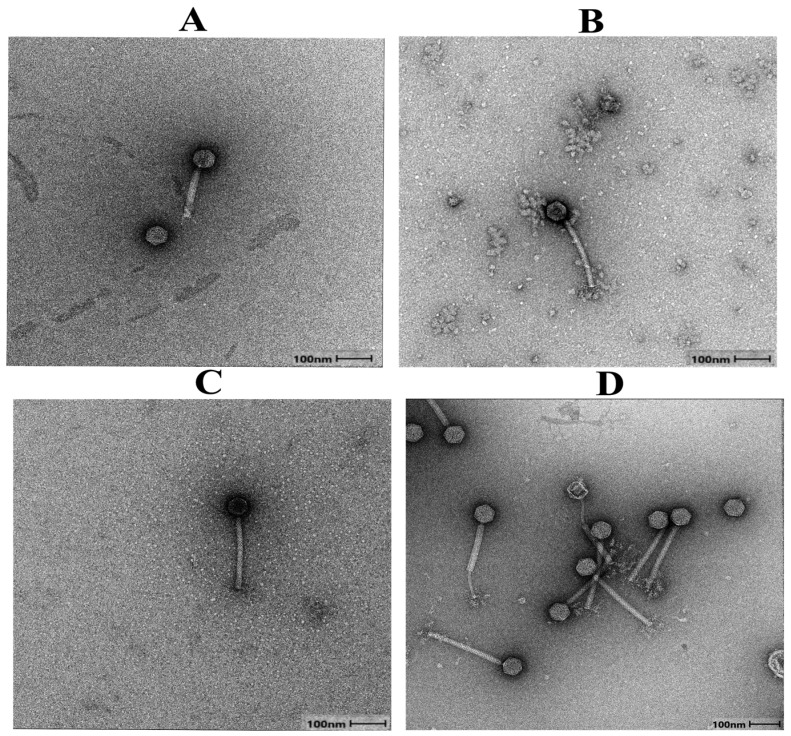
Transmission electron micrographs showing morphology of *Lactobacillus* phages induced by mitomycin C from isolated vaginal *L. crispatus* strains: (**A**) *L. crispatus* phage UC093; (**B**) *L. crispatus* phage UC101; (**C**) *L. crispatus* phage UC119; (**D**) *L. crispatus* phage UC164. Scale bars represent 100 nm.

Next, we exposed the four vaginal *L. crispatus* strains with predicted prophage regions to mitomycin C, a classical laboratory agent to induce phages [22,33,62,63], to screen for *in vitro* phage induction. Mitomycin C-based induction confirmed the presence of inducible phages in all four *L. crispatus* strains: UC0930205 (phage UC093), UC1010110 (phage UC101), UC1190127 (phage UC119), and UC1640140 (phage UC164). TEM showed that all the induced *L. crispatus* prophages belong to the *Caudoviricetes* class (tailed bacteriophages) with a typical myovirus morphology of an icosahedral head (~60 nm diameter) and a relatively thick contractile tail (Figure 1), which closely resembled the characteristic tail-to-head diameter ratio observed in induced *Lactobacillus* phages from other studies [18,22,62].

### 3.2. Genome Characterization of L. crispatus Phages

**Figure 2:**
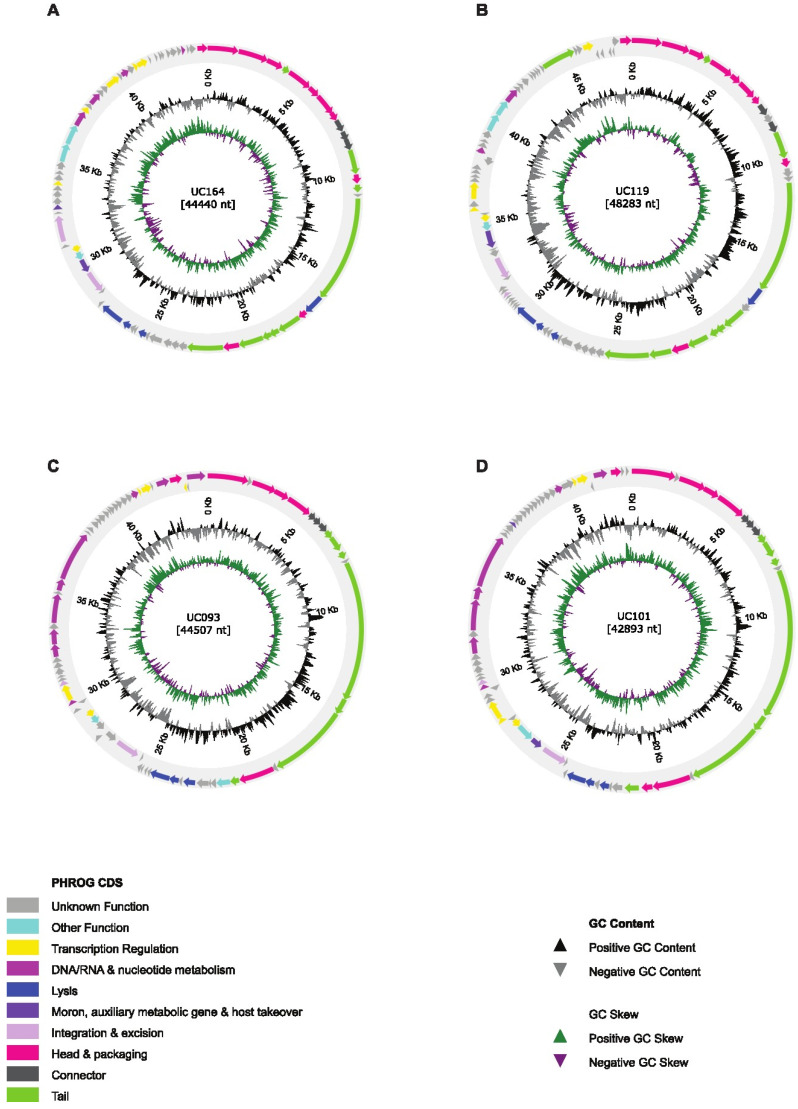
Genome organization of *L. crispatus* phages: (**A**) UC164, (**B**) UC119, (**C**) UC093 and (**D**) UC101. Functional gene annotations are color-coded. Predicted ORFs and their respective orientations are represented with arrows. GC content and GC skew (over- or under-abundance of guanine and cytosine in regions) are also shown.

DNA was extracted from the four mitomycin C-induced phages and whole-genome sequenced. Based on functional annotations, the predicted proteins were grouped into categories, including structure and assembly genes, cell lysis, integration and excision, among others, while hypothetical proteins with no clearly defined function in the core gene modules were grouped under “other functions or unknown functions” (Figure 2).

For all four phages, genome annotation revealed common core-encoded proteins, including those involved in head and packaging (terminase large and small subunits and head proteins), integration and lysis (integrase, endolysin and holin), and transcription regulation (transcription activator and antirepressor). However, only phages UC101 and UC164 had superinfection exclusion proteins (Table S1). The closest BLASTp hits to each of the CDSs in each phage genome are provided in Table S2. To identify prophage integration sites or loci within the bacterial host genome, the genome sequences of the induced prophages were aligned to their corresponding *L. crispatus* host genomes. The integrase genes of the induced phages were near the host’s bacterial XerC (bacterial tyrosine recombinase) gene, suggesting lysogenic lifestyles [64] (Figure S1). Similar genomic co-localization has been reported in transcription studies of temperate phages [65].

**Figure 3:**
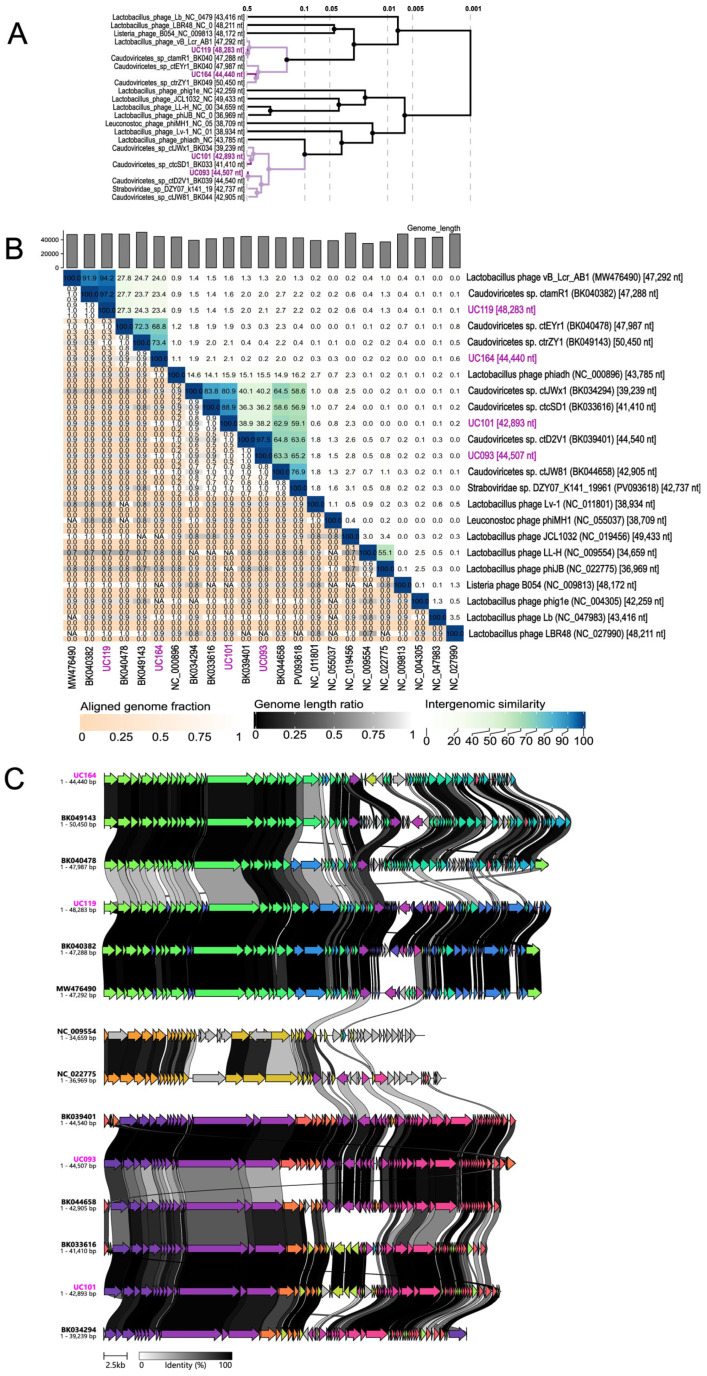
Comparative genomics of *L. crispatus* phages UC093, UC101, UC119, and UC164. (**A**) Phylogenetic tree constructed from viral proteome comparisons using ViPTree, showing the evolutionary relationship between the four *L. crispatus* phages and their closest genomic relatives (BLASTn hits with >90% nucleotide sequence identity). (**B**) Intergenomic similarity heatmap generated with VIRIDIC. Right half of the heatmap shows varying ranges of pairwise intergenomic similarities (%) and color gradients (darker hues = higher similarity). Phages identified in this study are highlighted in purple font. (**C**) Gene cluster comparison of induced phages with Clinker. Coding sequences are represented as arrows. Shaded regions between genomes are highlighted by amino acid ORF identity (0–100%), which indicates conserved gene blocks and variable phage gene regions. Each similarity group is assigned its unique color. Genome lengths of all phages are highlighted next to each phage.

To compare evolutionary relatedness, viral sequences from GenBank with >90% BLASTn similarity were uploaded to ViPTree and VIRIDIC. In the proteomic tree (Figure 3A), phages UC093, UC101, UC119, and UC164 clustered closely with *Caudoviricetes* species BK039401, BK033616, BK040382, and BK049143, respectively. Based on GenBank metadata, all these reference phages were recovered from the posterior fornix of female participants in a US-based human metagenome study [66]. Pairwise intergenomic comparisons with VIRIDIC (Figure 3B) revealed that phage UC119 shares 97.2% similarity with *Caudoviricetes* sp ctamR1 (BK040382). This indicates that they belong to the same species and potentially represents a conserved phage lineage within *L. crispatus* or other closely related vaginal *Lactobacillus* species. The same was observed for phage UC093, which had 97.5% similarity with *Caudoviricetes* sp ctD2V1 (BK039401). In contrast, phage UC164 shared only 73.4% similarity with *Caudoviricetes* sp ctrZY1 (BK049143) (Figure 3B), which is below the 95% species level threshold but above the proposed genus-level threshold of 70% [55]. This suggests that they are classified within the same genus but represent distinct species. Similarly, phage UC101 clustered most closely with *Caudoviricetes* sp ctcSD1 (BK033616) at 88.9% similarity, also suggesting that phage UC101 might be a distinct species (Figure 3B).

We further compared the phage gene clusters by uploading the four phage genomes, including references, to Clinker, with the minimum alignment sequence identity preset to 0.3 [56]. Phages UC093, UC101, UC119, and UC164 had the most gene synteny or conserved gene regions with *Caudoviricetes* reference genomes BK039401, BK033616, BK040382, and BK049143, respectively, as indicated by the amino acid identity of the open reading frames (ORFs) (Figure 3C). These patterns are consistent with the phylogenetic observations in Figure 3A,B in terms of phage genome relatedness to their closest reference genomes. The genomes of all the induced phages were further aligned to predicted prophage sequences of medium–high quality in the respective host genomes (Table S3).

### 3.3. In Vitro Assessment of Phage-like Particles and Bacterial Growth Following Copper Ion and Tenofovir Exposure

To explore whether *in vitro* exposure of vaginally derived *L. crispatus* strains to clinically relevant compounds is associated with the appearance of phage-like particles, we exposed bacterial cultures to physiologically relevant concentrations of copper ions (2.0–3.0 × 10^−6^ M) [28,57,58] and TFV (500–8000 ng/mL) [32,59,60,61]. These compounds were selected based on their widespread use in women’s sexual health and reported associations with vaginal *L. crispatus* depletion and potential antiviral activity, respectively [28,29,30,32].

**Figure 4:**
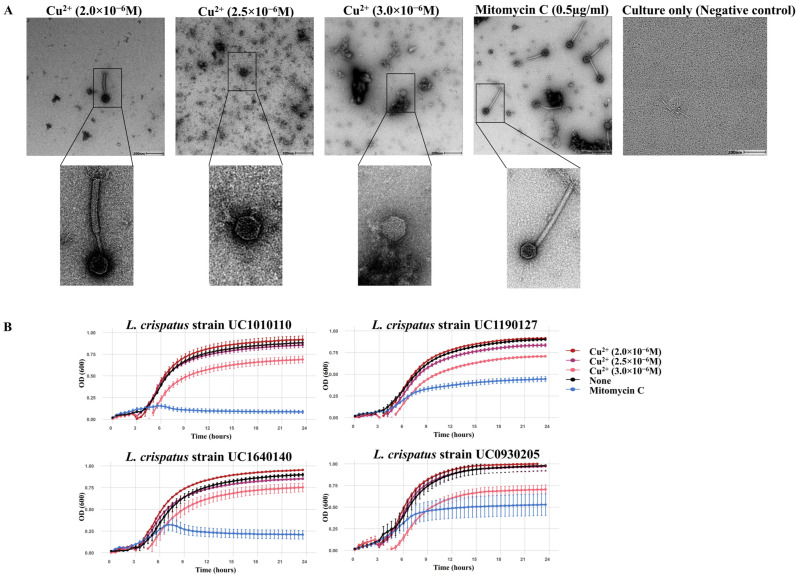
*L. crispatus* prophage induction using copper ions. (**A**) TEM images showing morphology of phage-like structures induced by various concentrations of copper ions (Cu^2+^), positive control (mitomycin C) and negative control (bacterial culture without Cu^2+^ or mitomycin C) from *L. crispatus* strain UC1640140. Scale bars represent 200 nm. (**B**) Growth kinetics of vaginal *L. crispatus* strains (UC1010110, UC1190127, UC1640140, and UC0930205) over 24 h in the presence of 2.0–3.0 × 10^−6^ M copper ions, mitomycin C (0.5 µg/mL) or no additions (no Cu^2+^, no mitomycin C). Background (media-only control) was subtracted. Error bars represent SEM (from 3 biological replicates).

Exposure of *L. crispatus* during the early log phase (3 h post-incubation) to copper ion concentrations comparable to those previously reported in vaginal fluid [57,58] (2.0–3.0 × 10^−6^ M) was associated with the appearance of phage-like particles by TEM, suggesting prophage induction (Figure 4A). At the lowest concentration tested (2.0 × 10^−6^ M), intact phage particles were observed (Figure 4A), although their morphology differed from that of phage particles induced with mitomycin C in the same *L. crispatus* strain (Figure 4A). At higher concentrations (2.5–3.0 × 10^−6^ M), only capsid-like structures were observed by TEM. The growth kinetics of the four *L. crispatus* strains in the presence of copper ions showed that growth was modestly inhibited at 3.0 × 10^−6^ M, while growth at the lower concentrations tested was comparable to untreated controls (Figure 4B). These observations suggest that exposure to low physiologically relevant copper concentrations can be associated with the presence of phage-like particles without substantial effects on host growth, while higher concentrations coincide with reduced growth and structurally incomplete phage-like particles.

**Figure 5:**
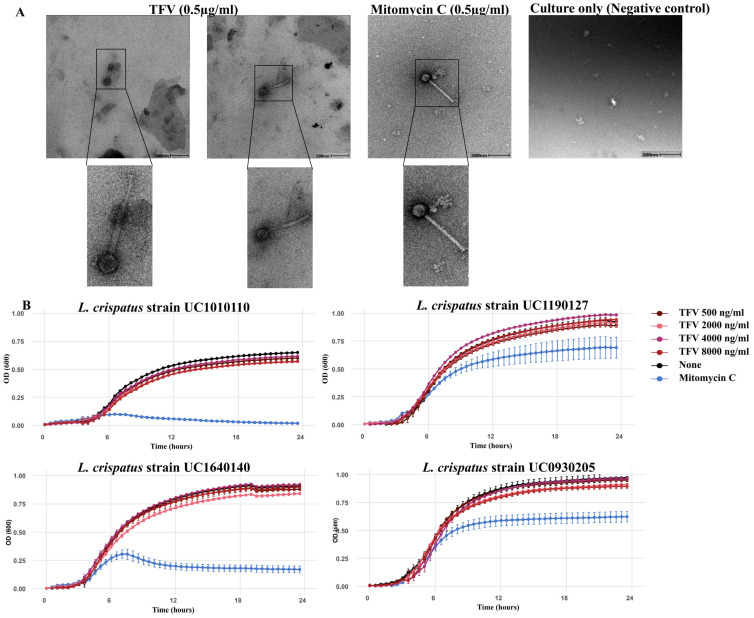
*L. crispatus* prophage induction using tenofovir. (**A**) Morphology of phage-like structures after exposure to TFV (500 ng/mL) and mitomycin C in *L. crispatus* strain UC1640140, visualized using TEM. Positive (mitomycin C) and negative controls (no mitomycin C, no TFV, bacteria only) were included. Scale bars represent 200 nm. (**B**) Growth kinetics of vaginal *L. crispatus* strains (UC1010110, UC1190127, UC1640140, and UC0930205) over a 24-h duration in the presence of physiologically relevant tenofovir concentrations (500–8000 ng/mL), mitomycin C (0.5 µg/mL) or no additions (none). Background (media-only control) was subtracted. Error bars represent SEM (from 3 biological replicates).

Exposure of *L. crispatus* strains to a range of relevant pharmacokinetic concentrations of TFV (500–8000 ng/mL) [32,59,60,61] during the early log phase resulted in the observation by TEM of phage-like particles only at the lowest concentration tested (500 ng/mL, Figure 5A), which is similar to the concentrations detected in vaginal fluid after oral dosing [61]. The growth kinetics were similar in TFV-exposed versus unexposed cultures across all the concentrations tested (Figure 5B).

## 4. Discussion

This study provides new insights into the genomic diversity of vaginal *Lactobacillus crispatus* prophages through the identification and comparative genome analysis of inducible prophages from *L. crispatus* isolates among South African women. Annotation and comparison of the four phage genomes to viral reference genomes in GenBank revealed that the genome sizes were consistent with the previously reported size range of temperate phage genomes found in *Lactobacillus* species [18,67,68]. All the phage genomes encoded hallmark genes for integration, excision, and host lysis. While there were largely common core proteins among the phages, notable differences were identified, including the presence of a superinfection exclusion (Sie) gene in phages UC101 and UC164 only. This gene has been shown to play a role in phage defense [69] and prevents lysogens from co-infection of the same or related phages [70,71,72]. To our knowledge, this is the first report of a Sie gene in an inducible *L. crispatus* phage, with previous evidence limited to non-*crispatus Lactobacillus* species [65,73,74]. Additionally, all the phage–bacterial host integration sites revealed a tyrosine recombinase integrase adjacent to the attachment sites, supporting a conserved mechanism of site-specific recombination, as seen in other studies on temperate phages [64,75].

Phylogenomic comparisons with ViPTree, VIRIDIC, and Clinker showed differences in the evolutionary relatedness of our phages to reference phages. Phages UC093 and UC119 shared high intergenomic similarities (>97%) with reference *Caudoviricetes* species in GenBank, meeting the virus species-level classification thresholds (>95%) under the current International Committee on Taxonomy of Viruses (ICTV) and VIRIDIC guidelines [55,76], suggesting that these phages are part of a conserved lineage of *Lactobacillus*-infecting phages. On the other hand, phages UC101 and UC164 each shared less than 90% intergenomic similarity with their closest reference genomes, falling below the VIRIDIC species-level cutoff (95%), and, in the case of UC164, approaching the genus-level threshold of 70%. These results were consistent with the phage genome comparisons using Clinker, highlighting the genomic uniqueness and divergence of these phages and further underscoring the underrepresentation of vaginal-derived prophage sequences in the current databases. Interestingly, the closest viral reference genomes for all four phages (UC093, UC101, UC119 and UC164) originated from the vaginal fornix of a female participant in the same study [66]. This finding aligns with our recent viral metagenomic study on vaginal samples from South African adolescents [77] in which an unclassified *Caudoviricetes* genome shared high nucleotide identity with a previously published reference viral genome [66] from the FGT.

As a hypothesis-generating investigation, we examined whether exposure of vaginally derived *L. crispatus* strains to copper ions and tenofovir, selected for their frequent use by women in South Africa and reported associations with vaginal microbiota shifts, was associated with the appearance of phage-like particles. Copper ion exposure at concentrations reported in vaginal secretions from Cu-IUD users [57,58] coincided with the presence of phage-like particles observed by TEM, consistent with reports of copper-associated phage induction in other environmental systems [78,79]. However, as TEM-based visualization alone does not demonstrate prophage induction, these observations should be interpreted cautiously. At higher concentrations, incomplete phage-like particles and reduced bacterial growth were observed, consistent with the known antimicrobial effects of copper [80]. These findings suggest that elevated copper levels may be broadly detrimental to both bacterial hosts and phage particles, potentially through oxidative stress-related mechanisms, although prophage-specific effects cannot be distinguished from general toxicity in the absence of quantitative molecular assays.

Exposure to 500 ng/mL TFV, a concentration more similar to cervicovaginal concentrations following oral dosage of tenofovir disoproxil fumarate (TDF), was associated with the appearance of phage-like particles without affecting bacterial growth. While intracellular uptake and metabolism of TFV [81] could plausibly influence host physiological states that are permissive to prophage activation, such mechanisms remain speculative and warrant further investigation.

Overall, these findings are preliminary and hypothesis-generating. Definitive demonstration and characterization of prophage induction will require quantitative approaches, including molecular assays of phage activation, host-range testing using prophage-cured strains, and more physiologically relevant *in vitro* models incorporating vaginal bacterial consortia. Finally, the *L. crispatus* strains examined here represent a limited subset of the species’ diversity. Given the substantial variation in prophage content across *L. crispatus* genomes [18,21,82], induction-related responses are likely strain-dependent, highlighting the need for broader surveys to assess generalizability. Furthermore, while the focus on *L. crispatus* provides important foundational insight into prophage diversity and potential inducibility within this species, its relatively low prevalence in South African women and girls [83,84,85] may limit the generalizability of these findings across the broader vaginal microbiota landscapes characteristic of this population.

## 5. Conclusions

In this study, we identified and genomically characterized inducible prophages from vaginal *L. crispatus* isolates obtained from South African women, revealing distinct genome organization and phylogenetic diversity, with two candidate novel species. We further present hypothesis-generating observations linking *in vitro* exposure to physiologically relevant concentrations of copper ions and TFV with the appearance of phage-like particles. Formal demonstration and quantitative characterization of prophage induction, as well as evaluation of its potential relevance to reproductive health, will require further experimental investigation.

## Figures and Tables

**Table 1:** Predicted prophage sequences identified in four vaginal *Lactobacillus crispatus* genomes and their closest BLASTn viral hits. Regions classified as intact by PHASTER or medium–high-quality regions by CheckV are included.

Host Strain ID	Phage Contig ID	Genome Length (bp)	GC%	CheckV Quality	PHASTER Classification	Completeness	Query Coverage	% Nucleotide Identity	NCBI Closest Phage
UC0930205	NODE4	42,405	36.30%	Complete	Intact	100.00%	98.00%	98.79%	BK039401
	NODE17	31,532	43.10%	Medium	Incomplete	66.68%	73.00%	94.19%	BK038203
	NODE19	30,580	42.70%	Medium	Incomplete	64.66%	100.00%	99.75%	BK038203
UC1010110	NODE9	42,894	35.40%	Complete	Intact	100.00%	89.00%	96.85%	BK033616
	NODE4	51,736	39.30%	High	Incomplete	100.00%	72.00%	94.25%	BK049143
	NODE2	42,308	41.20%	Medium	Incomplete	89.46%	94.00%	97.59%	BK038203
	NODE32	23,956	35.80%	Medium	Not found	54.21%	100.00%	100.00%	BK036340
UC1190127	NODE1	38,203	39.60%	High	Intact	100.00%	87.00%	99.97%	BK040382
UC1640140	NODE7	39,584	40.00%	Medium	Incomplete	83.70%	86.00%	94.20%	BK040478

## Data Availability

Bacterial host genome sequences of *L. crispatus* strains were submitted to ENA under project PRJEB104024 with accession numbers ERS27360631, ERS27360632, ERS27360633 and ERS27360634. Complete genome sequences of *L. crispatus* phages UC119, UC164, UC101, and UC093 were submitted to the NCBI GenBank database under accession numbers PX260543, PX260544, PX260545 and PX260546, respectively.

## Supplementary Materials

The following supporting information can be downloaded at: https://www.mdpi.com/article/10.3390/v18050519/s1, Figure S1: Schematic of phage–bacterial host alignment showing integration sites or flanking bacterial genome. Table S1: Genome annotations identified in *L. crispatus* phages UC119, UC093, UC101, and UC164. Table S2: Functional characterization of ORFs and closest BLASTP hits of *L. crispatus* phages UC093, UC101, UC119, and UC164. Table S3: Alignment of induced phage genomes to predicted prophage regions in corresponding *L. crispatus* host strains.

## Abbreviations

The following abbreviations are used in this manuscript:

BV	Bacterial vaginosis
Cu-IUD	Copper intrauterine device
FGT	Female genital tract
HIV	Human immunodeficiency virus
ICTV	International Committee on Taxonomy of Viruses
LMICs	Low- and middle-income countries
ORFs	Open reading frames
PrEP	Pre-exposure prophylaxis
Sie	Superinfection exclusion
STIs	Sexually transmitted infections
TEM	Transmission electron microscopy
TFV	Tenofovir
