# Supplementary material for: Genomic Diversity of Vaginal Lactobacillus crispatus Prophages from South African Women
Source: Viruses. 2026 Apr 30;18(5):519. doi: 10.3390/v18050519 (PMC13211564; doi:10.3390/v18050519)
Supplement: Supplementary file 1 [file viruses-18-00519-s001.zip › viruses-4244507-supplementary.pdf]

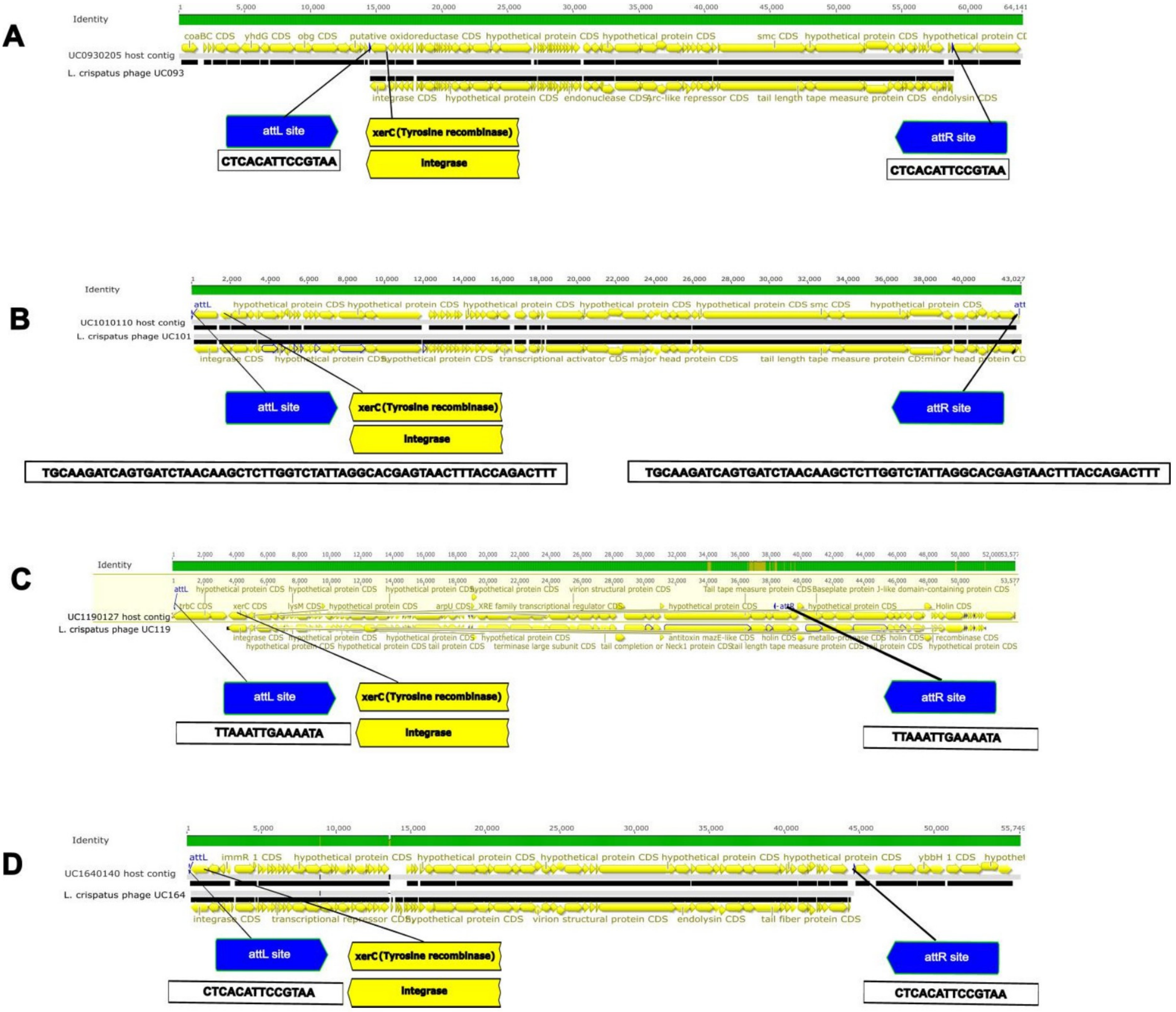

Supplementary Figure S1: Schematic of phage-bacterial host alignment showing integration sites or flanking bacterial genome regions for:

(A) *L. crispatus* phage UC093 showing integration between host gene xerC (tyrosine recombinase flanked by attL and attR sites with a 16 bp core sequence (CTAAAAAATGGCTAAA).

(B) *L. crispatus* phage UC101 is flanked by attL and attR sites with a 62bp core sequence (TGCAAGATCAGTGATCTAACAAGCTCTTGGTCTATTAGGCACGAGTAACTTTACCAGACTTT).

(C) *L. crispatus* phage UC119 is similarly flanked by attL and attR sites with a 14bp core sequence (TTAAATTGAAAATA)

(D) *L. crispatus* phage UC164 is similarly flanked by a 14 bp core sequence (CTCACATTCCGTAA) in the phage attachment sites (attL and attR)

Supplementary Table (S1): Genome annotations identified in *Lactobacillus crispatus* phages UC119, UC093, UC101, UC164

**Phage Name**	**Minimum**	**Maximum**	**Length**	**Direction**	**Type**	**Name**
UC119	11324	16801	5478	forward	CDS	tail length tape measure protein CDS
UC119	23313	25487	2175	forward	CDS	tail protein CDS
UC119	43739	45415	1677	forward	CDS	tail protein CDS
UC119	1	1473	1473	forward	CDS	terminase large subunit CDS
UC119	1486	2871	1386	forward	CDS	portal protein CDS
UC119	8776	10110	1335	forward	CDS	tail sheath CDS
UC119	4028	5239	1212	forward	CDS	head maturation protease CDS
UC119	18195	19322	1128	forward	CDS	baseplate hub CDS
UC119	29214	30335	1122	forward	CDS	endolysin CDS
UC119	22208	23296	1089	forward	CDS	tail fiber protein CDS
UC119	20310	21341	1032	forward	CDS	baseplate wedge subunit CDS
UC119	5790	6749	960	forward	CDS	major head protein CDS
UC119	16813	17772	960	forward	CDS	endolysin CDS
UC119	39614	40492	879	forward	CDS	DNA methyltransferase CDS
UC119	21331	22179	849	forward	CDS	structural protein CDS
UC119	36175	36993	819	forward	CDS	anti-repressor Ant CDS
UC119	2876	3661	786	forward	CDS	head morphogenesis CDS
UC119	40495	41280	786	forward	CDS	recombinase CDS
UC119	41282	42016	735	forward	CDS	DnaD-like helicase loader CDS
UC119	7224	7820	597	forward	CDS	tail completion or Neck1 protein CDS
UC119	27134	27730	597	forward	CDS	hypothetical protein CDS
UC119	47715	4	573	forward	CDS	terminase small subunit CDS
UC119	8212	8772	561	forward	CDS	tail terminator CDS
UC119	5239	5775	537	forward	CDS	virion structural protein CDS
UC119	45887	46390	504	forward	CDS	transcriptional activator CDS

UC119	19335	19808	474	forward	CDS	baseplate spike CDS
UC119	10615	11061	447	forward	CDS	hypothetical protein CDS
UC119	10125	10556	432	forward	CDS	virion structural protein CDS
UC119	25510	25932	423	forward	CDS	hypothetical protein CDS
UC119	7830	8249	420	forward	CDS	hypothetical protein CDS
UC119	17785	18195	411	forward	CDS	hypothetical protein CDS
UC119	19756	20163	408	forward	CDS	baseplate wedge subunit CDS
UC119	27875	28276	402	forward	CDS	holin CDS
UC119	28744	29142	399	forward	CDS	holin CDS
UC119	6766	7152	387	forward	CDS	virion structural protein CDS
UC119	25926	26312	387	forward	CDS	hypothetical protein CDS
UC119	26656	27039	384	forward	CDS	hypothetical protein CDS
UC119	39246	39611	366	forward	CDS	hypothetical protein CDS
UC119	3672	4028	357	forward	CDS	tail protein CDS
UC119	26309	26662	354	forward	CDS	hypothetical protein CDS
UC119	42487	42810	324	forward	CDS	hypothetical protein CDS
UC119	47345	47668	324	forward	CDS	hypothetical protein CDS
UC119	38443	38742	300	forward	CDS	HTH DNA binding protein CDS
UC119	35794	36087	294	forward	CDS	hypothetical protein CDS
UC119	28487	28744	258	forward	CDS	hypothetical protein CDS
UC119	45591	45839	249	forward	CDS	hypothetical protein CDS
UC119	11033	11278	246	forward	CDS	hypothetical protein CDS
UC119	38747	38992	246	forward	CDS	hypothetical protein CDS
UC119	37636	37872	237	forward	CDS	hypothetical protein CDS
UC119	35575	35793	219	forward	CDS	transcriptional repressor CDS
UC119	37006	37224	219	forward	CDS	hypothetical protein CDS
UC119	37423	37635	213	forward	CDS	hypothetical protein CDS

UC119	28294	28503	210	forward	CDS	hypothetical protein CDS
UC119	42013	42222	210	forward	CDS	hypothetical protein CDS
UC119	42233	42424	192	forward	CDS	hypothetical protein CDS
UC119	31459	31644	186	forward	CDS	hypothetical protein CDS
UC119	43070	43255	186	forward	CDS	hypothetical protein CDS
UC119	31106	31288	183	forward	CDS	excisionase CDS
UC119	27733	27912	180	forward	CDS	hypothetical protein CDS
UC119	45428	45604	177	forward	CDS	hypothetical protein CDS
UC119	37221	37388	168	forward	CDS	hypothetical protein CDS
UC119	31301	31459	159	forward	CDS	hypothetical protein CDS
UC119	30775	30930	156	forward	CDS	hypothetical protein CDS
UC119	39001	39156	156	forward	CDS	hypothetical protein CDS
UC119	43587	43742	156	forward	CDS	hypothetical protein CDS
UC119	30621	30770	150	forward	CDS	hypothetical protein CDS
UC119	43380	43511	132	forward	CDS	hypothetical protein CDS
UC119	30364	30492	129	forward	CDS	hypothetical protein CDS
UC119	43255	43380	126	forward	CDS	hypothetical protein CDS
UC119	42941	43054	114	forward	CDS	hypothetical protein CDS
UC119	30489	30593	105	forward	CDS	hypothetical protein CDS
UC119	30984	31079	96	forward	CDS	hypothetical protein CDS
UC119	31842	32990	1149	reverse	CDS	integrase CDS
UC119	33580	34494	915	reverse	CDS	antitoxin mazE-like CDS
UC119	34507	34959	453	reverse	CDS	metallo-protease CDS
UC119	37937	38353	417	reverse	CDS	hypothetical protein CDS
UC119	34968	35336	369	reverse	CDS	transcriptional regulator CDS
UC119	33077	33418	342	reverse	CDS	hypothetical protein CDS
UC119	46642	46812	171	reverse	CDS	hypothetical protein CDS

UC119	31727	31876	150	reverse	CDS	hypothetical protein CDS
UC119	46408	46548	141	reverse	CDS	hypothetical protein CDS
UC119	47072	47212	141	reverse	CDS	hypothetical protein CDS
UC119	35323	35445	123	reverse	CDS	hypothetical protein CDS
UC119	47268	47375	108	reverse	CDS	hypothetical protein CDS
UC119	33425	33529	105	reverse	CDS	hypothetical protein CDS
UC093	7971	14471	6501	forward	CDS	tail length tape measure protein CDS
UC093	15231	18944	3714	forward	CDS	tail protein CDS
UC093	35759	38086	2328	forward	CDS	DNA primase CDS
UC093	1	1896	1896	forward	CDS	terminase large subunit CDS
UC093	19139	20779	1641	forward	CDS	minor head protein CDS
UC093	33682	35031	1350	forward	CDS	DNA helicase CDS
UC093	3920	5122	1203	forward	CDS	major head protein CDS
UC093	2095	3237	1143	forward	CDS	portal protein CDS
UC093	24039	24962	924	forward	CDS	endolysin CDS
UC093	43555	44406	852	forward	CDS	HNH endonuclease CDS
UC093	14461	15231	771	forward	CDS	distal tail protein Dit CDS
UC093	30019	30768	750	forward	CDS	anti-repressor Ant CDS
UC093	6601	7311	711	forward	CDS	major tail protein CDS
UC093	3200	3907	708	forward	CDS	head maturation protease CDS
UC093	32693	33400	708	forward	CDS	Sak4-like ssDNA annealing protein CDS
UC093	21220	21861	642	forward	CDS	PblB-type receptor binding CDS
UC093	42117	42710	594	forward	CDS	HNH endonuclease CDS
UC093	32125	32700	576	forward	CDS	HNH endonuclease CDS
UC093	42753	43319	567	forward	CDS	terminase small subunit CDS
UC093	22209	22766	558	forward	CDS	hypothetical protein CDS
UC093	22833	23387	555	forward	CDS	holin CDS

UC093	35196	35747	552	forward	CDS	single strand DNA binding protein CDS
UC093	41382	41846	465	forward	CDS	transcriptional activator CDS
UC093	23594	24049	456	forward	CDS	holin CDS
UC093	5799	6236	438	forward	CDS	tail completion or Neck1 protein CDS
UC093	20797	21219	423	forward	CDS	tail fiber protein CDS
UC093	6233	6619	387	forward	CDS	tail protein CDS
UC093	5456	5806	351	forward	CDS	head closure Hc1 CDS
UC093	7404	7751	348	forward	CDS	tail protein CDS
UC093	5143	5484	342	forward	CDS	head-tail adaptor Ad1 CDS
UC093	40863	41201	339	forward	CDS	endonuclease CDS
UC093	31290	31616	327	forward	CDS	hypothetical protein CDS
UC093	39036	39329	294	forward	CDS	hypothetical protein CDS
UC093	40327	40611	285	forward	CDS	hypothetical protein CDS
UC093	33400	33681	282	forward	CDS	hypothetical protein CDS
UC093	31609	31884	276	forward	CDS	hypothetical protein CDS
UC093	40601	40873	273	forward	CDS	hypothetical protein CDS
UC093	38626	38889	264	forward	CDS	hypothetical protein CDS
UC093	21866	22126	261	forward	CDS	hypothetical protein CDS
UC093	31877	32125	249	forward	CDS	hypothetical protein CDS
UC093	40085	40327	243	forward	CDS	hypothetical protein CDS
UC093	18955	19185	231	forward	CDS	hypothetical protein CDS
UC093	39657	39887	231	forward	CDS	hypothetical protein CDS
UC093	39880	40110	231	forward	CDS	hypothetical protein CDS
UC093	30771	30989	219	forward	CDS	excisionase and transcriptional regulator CDS
UC093	39343	39558	216	forward	CDS	hypothetical protein CDS
UC093	27796	27993	198	forward	CDS	hypothetical protein CDS
UC093	38332	38526	195	forward	CDS	hypothetical protein CDS

UC093	1886	2077	192	forward	CDS	hypothetical protein CDS
UC093	29812	30003	192	forward	CDS	DNA binding protein CDS
UC093	25436	25615	180	forward	CDS	hypothetical protein CDS
UC093	38879	39049	171	forward	CDS	hypothetical protein CDS
UC093	23433	23597	165	forward	CDS	hypothetical protein CDS
UC093	29402	29566	165	forward	CDS	hypothetical protein CDS
UC093	7813	7965	153	forward	CDS	hypothetical protein CDS
UC093	30986	31138	153	forward	CDS	hypothetical protein CDS
UC093	24955	25101	147	forward	CDS	hypothetical protein CDS
UC093	35028	35174	147	forward	CDS	hypothetical protein CDS
UC093	25129	25248	120	forward	CDS	hypothetical protein CDS
UC093	41216	41335	120	forward	CDS	Arc-like repressor CDS
UC093	31150	31266	117	forward	CDS	hypothetical protein CDS
UC093	22108	22212	105	forward	CDS	hypothetical protein CDS
UC093	25332	25436	105	forward	CDS	hypothetical protein CDS
UC093	39560	39664	105	forward	CDS	hypothetical protein CDS
UC093	38162	38263	102	forward	CDS	hypothetical protein CDS
UC093	41843	41932	90	forward	CDS	hypothetical protein CDS
UC093	25843	27024	1182	reverse	CDS	integrase CDS
UC093	27172	27717	546	reverse	CDS	hypothetical protein CDS
UC093	27977	28384	408	reverse	CDS	hypothetical protein CDS
UC093	28387	28791	405	reverse	CDS	metallo-protease CDS
UC093	28794	29138	345	reverse	CDS	transcriptional regulator CDS
UC093	29588	29749	162	reverse	CDS	hypothetical protein CDS
UC093	43331	43468	138	reverse	CDS	Arc-like repressor CDS
UC093	25554	25655	102	reverse	CDS	hypothetical protein CDS
UC093	43489	43578	90	reverse	CDS	hypothetical protein CDS

UC101	8131	14649	6519	forward	CDS	tail length tape measure protein CDS
UC101	15412	18738	3327	forward	CDS	tail protein CDS
UC101	34059	36380	2322	forward	CDS	DNA primase CDS
UC101	1	1902	1902	forward	CDS	terminase large subunit CDS
UC101	18892	20541	1650	forward	CDS	minor head protein CDS
UC101	32131	33474	1344	forward	CDS	DNA helicase CDS
UC101	3958	5268	1311	forward	CDS	major head protein CDS
UC101	2089	3270	1182	forward	CDS	portal protein CDS
UC101	23473	24318	846	forward	CDS	endolysin CDS
UC101	28139	28936	798	forward	CDS	anti-repressor CDS
UC101	31127	31849	723	forward	CDS	Sak4-like ssDNA annealing protein CDS
UC101	14696	15412	717	forward	CDS	distal tail protein Dit CDS
UC101	3233	3943	711	forward	CDS	head maturation protease CDS
UC101	6765	7469	705	forward	CDS	major tail protein CDS
UC101	21144	21758	615	forward	CDS	tail protein CDS
UC101	41241	41831	591	forward	CDS	HNH endonuclease CDS
UC101	33477	34046	570	forward	CDS	single strand DNA binding protein CDS
UC101	39797	40333	537	forward	CDS	hypothetical protein CDS
UC101	20555	21028	474	forward	CDS	virion structural protein CDS
UC101	40504	40968	465	forward	CDS	transcriptional activator CDS
UC101	21857	22315	459	forward	CDS	hypothetical protein CDS
UC101	41992	42441	450	forward	CDS	terminase small subunit CDS
UC101	5957	6397	441	forward	CDS	tail completion or Neck1 protein CDS
UC101	22436	22852	417	forward	CDS	holin CDS
UC101	23085	23480	396	forward	CDS	holin CDS
UC101	6394	6783	390	forward	CDS	tail protein CDS
UC101	5286	5648	363	forward	CDS	head-tail adaptor Ad1 CDS

UC101	5614	5964	351	forward	CDS	head closure Hc1 CDS
UC101	7569	7913	345	forward	CDS	tail protein CDS
UC101	39472	39810	339	forward	CDS	endonuclease CDS
UC101	30292	30618	327	forward	CDS	hypothetical protein CDS
UC101	36792	37094	303	forward	CDS	hypothetical protein CDS
UC101	37397	37690	294	forward	CDS	hypothetical protein CDS
UC101	38939	39223	285	forward	CDS	hypothetical protein CDS
UC101	31849	32130	282	forward	CDS	hypothetical protein CDS
UC101	30611	30886	276	forward	CDS	hypothetical protein CDS
UC101	39213	39482	270	forward	CDS	hypothetical protein CDS
UC101	30879	31127	249	forward	CDS	hypothetical protein CDS
UC101	38703	38939	237	forward	CDS	hypothetical protein CDS
UC101	28939	29169	231	forward	CDS	hypothetical protein CDS
UC101	22868	23095	228	forward	CDS	hypothetical protein CDS
UC101	38313	38540	228	forward	CDS	hypothetical protein CDS
UC101	37922	38140	219	forward	CDS	hypothetical protein CDS
UC101	29773	29988	216	forward	CDS	excisionase and transcriptional regulator CDS
UC101	37704	37919	216	forward	CDS	hypothetical protein CDS
UC101	27926	28138	213	forward	CDS	transcriptional repressor CDS
UC101	1892	2089	198	forward	CDS	hypothetical protein CDS
UC101	29565	29756	192	forward	CDS	DNA binding protein CDS
UC101	18741	18926	186	forward	CDS	hypothetical protein CDS
UC101	29156	29341	186	forward	CDS	hypothetical protein CDS
UC101	7945	8127	183	forward	CDS	hypothetical protein CDS
UC101	36480	36662	183	forward	CDS	hypothetical protein CDS
UC101	38137	38316	180	forward	CDS	hypothetical protein CDS
UC101	37240	37410	171	forward	CDS	hypothetical protein CDS

UC101	29988	30140	153	forward	CDS	hypothetical protein CDS
UC101	22315	22464	150	forward	CDS	hypothetical protein CDS
UC101	37081	37227	147	forward	CDS	Lar-like restriction alleviation protein CDS
UC101	38541	38687	147	forward	CDS	hypothetical protein CDS
UC101	40314	40457	144	forward	CDS	Arc-like repressor CDS
UC101	24362	24493	132	forward	CDS	hypothetical protein CDS
UC101	42438	42569	132	forward	CDS	hypothetical protein CDS
UC101	42640	42771	132	forward	CDS	hypothetical protein CDS
UC101	30152	30268	117	forward	CDS	hypothetical protein CDS
UC101	36679	36774	96	forward	CDS	hypothetical protein CDS
UC101	24614	25780	1167	reverse	CDS	integrase CDS
UC101	26519	27322	804	reverse	CDS	metallo-protease CDS
UC101	25937	26470	534	reverse	CDS	superinfection exclusion CDS
UC101	27324	27692	369	reverse	CDS	transcriptional regulator CDS
UC101	29289	29501	213	reverse	CDS	hypothetical protein CDS
UC101	27694	27870	177	reverse	CDS	hypothetical protein CDS
UC101	40981	41100	120	reverse	CDS	hypothetical protein CDS
UC101	24514	24627	114	reverse	CDS	hypothetical protein CDS
UC164	41780	41850	71	forward	tRNA	tRNA
UC164	11131	16206	5076	forward	CDS	tail length tape measure protein CDS
UC164	21498	23189	1692	forward	CDS	tail fiber protein CDS
UC164	1	1458	1458	forward	CDS	terminase large subunit CDS
UC164	1474	2895	1422	forward	CDS	portal protein CDS
UC164	31259	32500	1242	forward	CDS	transposase CDS
UC164	4072	5262	1191	forward	CDS	head maturation protease CDS
UC164	19564	20745	1182	forward	CDS	baseplate wedge subunit CDS
UC164	8803	9972	1170	forward	CDS	tail sheath CDS

UC164	17600	18727	1128	forward	CDS	baseplate hub CDS
UC164	26550	27671	1122	forward	CDS	endolysin CDS
UC164	5828	6868	1041	forward	CDS	major head protein CDS
UC164	16218	17177	960	forward	CDS	endolysin CDS
UC164	35946	36905	960	forward	CDS	recombinase CDS
UC164	35071	35946	876	forward	CDS	DNA methyltransferase CDS
UC164	2879	3676	798	forward	CDS	head morphogenesis CDS
UC164	20735	21487	753	forward	CDS	structural protein CDS
UC164	36907	37653	747	forward	CDS	replication initiation protein CDS
UC164	39283	39972	690	forward	CDS	anti-repressor CDS
UC164	24422	25048	627	forward	CDS	hypothetical protein CDS
UC164	38152	38757	606	forward	CDS	DNA repair protein CDS
UC164	7259	7855	597	forward	CDS	tail completion or Neck1 protein CDS
UC164	5262	5828	567	forward	CDS	virion structural protein CDS
UC164	8258	8803	546	forward	CDS	tail terminator CDS
UC164	41009	41518	510	forward	CDS	transcriptional activator CDS
UC164	43957	14	498	forward	CDS	terminase small subunit CDS
UC164	18740	19213	474	forward	CDS	baseplate spike CDS
UC164	7873	8328	456	forward	CDS	head closure Hc1 CDS
UC164	6822	7259	438	forward	CDS	virion structural protein CDS
UC164	9991	10422	432	forward	CDS	virion structural protein CDS
UC164	19158	19574	417	forward	CDS	baseplate wedge subunit CDS
UC164	17190	17600	411	forward	CDS	virion structural protein CDS
UC164	26053	26451	399	forward	CDS	holin CDS
UC164	23208	23600	393	forward	CDS	hypothetical protein CDS
UC164	25218	25607	390	forward	CDS	holin CDS
UC164	23944	24327	384	forward	CDS	hypothetical protein CDS

UC164	40145	40525	381	forward	CDS	endonuclease CDS
UC164	34701	35078	378	forward	CDS	hypothetical protein CDS
UC164	10479	10847	369	forward	CDS	tail protein XkdN-like CDS
UC164	23597	23950	354	forward	CDS	hypothetical protein CDS
UC164	3721	4068	348	forward	CDS	tail length tape measure protein CDS
UC164	33044	33373	330	forward	CDS	hypothetical protein CDS
UC164	42675	42992	318	forward	CDS	hypothetical protein CDS
UC164	33385	33699	315	forward	CDS	hypothetical protein CDS
UC164	40522	40824	303	forward	CDS	hypothetical protein CDS
UC164	43588	43887	300	forward	CDS	hypothetical protein CDS
UC164	34167	34457	291	forward	CDS	hypothetical protein CDS
UC164	37653	37931	279	forward	CDS	transcriptional regulator CDS
UC164	39009	39272	264	forward	CDS	hypothetical protein CDS
UC164	38759	39004	246	forward	CDS	hypothetical protein CDS
UC164	33696	33935	240	forward	CDS	hypothetical protein CDS
UC164	25823	26053	231	forward	CDS	hypothetical protein CDS
UC164	32799	33029	231	forward	CDS	plasmid antitoxin with HTH domain CDS
UC164	37935	38147	213	forward	CDS	hypothetical protein CDS
UC164	33945	34154	210	forward	CDS	transcriptional repressor CDS
UC164	43005	43208	204	forward	CDS	hypothetical protein CDS
UC164	40824	41018	195	forward	CDS	transcriptional repressor CDS
UC164	25067	25249	183	forward	CDS	hypothetical protein CDS
UC164	34465	34644	180	forward	CDS	hypothetical protein CDS
UC164	42492	42671	180	forward	CDS	hypothetical protein CDS
UC164	43212	43385	174	forward	CDS	ParB-like partition nuclease CDS
UC164	39987	40142	156	forward	CDS	hypothetical protein CDS
UC164	27765	27917	153	forward	CDS	hypothetical protein CDS

UC164	42113	42265	153	forward	CDS	hypothetical protein CDS
UC164	10915	11061	147	forward	CDS	hypothetical protein CDS
UC164	30963	31106	144	forward	CDS	hypothetical protein CDS
UC164	25708	25839	132	forward	CDS	hypothetical protein CDS
UC164	42240	42365	126	forward	CDS	hypothetical protein CDS
UC164	41921	42031	111	forward	CDS	hypothetical protein CDS
UC164	43468	43575	108	forward	CDS	hypothetical protein CDS
UC164	32593	32694	102	forward	CDS	hypothetical protein CDS
UC164	41612	41701	90	forward	CDS	hypothetical protein CDS
UC164	28249	29361	1113	reverse	CDS	integrase CDS
UC164	29421	30110	690	reverse	CDS	superinfection exclusion CDS
UC164	30121	30525	405	reverse	CDS	metallo-protease CDS
UC164	30537	30878	342	reverse	CDS	transcriptional regulator CDS
UC164	28131	28259	129	reverse	CDS	hypothetical protein CDS

Supplementary Table S2

Functional characterization of ORFs and closest BLASTP hits of *L crispatus* phages UC093, UC101, UC119, UC164

Functional Group	Function	Phage	BLASTP Best Match	Percentage Identity	GenBank Accession Number
**Structure and Assembly**	**Terminase large subunit**	*L. crispatus* phage UC093 [1 – 1896]	phage terminase large subunit [*Lactobacillus crispatus*]	100.00%	WP_060808454.1
		L. crispatus phage UC101 [1 – 1902]	terminase large subunit [*Lactobacillus helveticus*]	92.62%	WP_216695231.1
		L. crispatus phage UC119 [1 – 1473]	phage terminase large subunit [*Lactobacillus crispatus*]	89.67%	WP_269256605.1
		L. crispatus phage UC164 [1 – 1458]	phage terminase large subunit [*Lactobacillus crispatus*]	99.79%	WP_133467637.1
	**Terminase small subunit**	*L. crispatus* phage UC093 [42753 – 43319]	phage terminase small subunit P27 family [*Lactobacillus crispatus*]	99.33%	WP_005725729.1
		L. crispatus phage UC101 [41992 – 42441]	phage terminase small subunit P27 family [*Lactobacillus crispatus*]	99.33%	WP_060464056.1
		L. crispatus phage UC119 [47715 - 4]	terminase small subunit [*Lactobacillus crispatus*]	95.51%	WP_089150821.1
		L. crispatus phage UC164 [43957 – 14]	hypothetical protein [*Lactobacillus crispatus*]	98.76%	WP_353579913.1
	**Portal protein**	*L. crispatus* phage UC093 [2095 – 3237]	phage portal protein [*Lactobacillus crispatus*]	100.00%	WP_340858868.1
		L. crispatus phage UC101 [2089 – 3270]	phage portal protein [*Lactobacillus crispatus*]	100.00%	WP_005725737.1
		L. crispatus phage UC119 [1486 – 2871]	phage portal protein [*Lactobacillus* sp. B4026]	55.91%	WP_267413677.1
		L. crispatus phage UC164 [1474 – 2895]	phage portal protein [uncultured *Lactobacillus* sp.]	38.84%	WP_294829783.1
	**Head morphogenesis**	*L. crispatus* phage UC093	-	-	-
		L. crispatus phage UC101	-	-	-
		L. crispatus phage UC119 [2876 – 3661]	phage head morphogenesis protein [*Lactobacillus crispatus*]	100.00%	WP_005719013.1
		L. crispatus phage UC164 [2879 – 3676]	phage minor head protein [*Lactobacillus crispatus*]	96.99%	WP_133467611.1
	**Head maturation protease**	*L. crispatus* phage UC093 [3200 – 3907]	head maturation protease, ClpP-related [*Lactobacillus crispatus*]	98.72%	WP_285031657.1
		L. crispatus phage UC101 [3233 -3943]	head maturation protease, ClpP-related [*Lactobacillus crispatus*]	95.76%	WP_005728814.1

		L. crispatus phage UC119 [4028 – 5239]	MAG: hypothetical protein [*Caudoviricetes* sp.]	100.00%	WP_319464633.1
		L. crispatus phage UC164 [4072 – 5262]	MAG: hypothetical protein [Bacteriophage sp.]	39.08%	QHJ77124.1
	**Major head protein**	*L. crispatus* phage UC093 [3920 – 5122]	phage major capsid protein [*Lactobacillus crispatus*]	99.75%	WP_340849977.1
		L. crispatus phage UC101 [3958 – 5268]	phage major capsid protein [*Lactobacillus crispatus*]	98.62%	WP_133472241.1
		L. crispatus phage UC119 [5790 – 6749]	major capsid family protein [*Lactobacillus crispatus*]	98.75%	WP_101891426.1
		L. crispatus phage UC164 [5858 – 6868]	major capsid family protein [*Lactobacillus crispatus*]	98.27%	WP_270028708.1
	**Virion structural protein**	*L. crispatus* phage UC093	-	-	-
		L. crispatus phage UC101 [20555 – 21028]	MAG TPA: hypothetical protein [*Caudoviricetes* sp.]	88.54%	DAS15042.1
		L. crispatus phage UC119 [positions 5239-5775]	MAG: hypothetical protein [Bacteriophage sp.]	53.33%	QHJ85257.1
		L. crispatus phage UC119 [6766 – 7152]	MAG: hypothetical protein [Bacteriophage sp.]	31.03%	QHJ77121.1
		L. crispatus phage UC119 [positions 10125-10556]	MAG: hypothetical protein [Bacteriophage sp.]	40.32%	QHJ77116.1
		L. crispatus phage UC164 [positions 5262 – 5858]	MAG: hypothetical protein [Bacteriophage sp.]	34.46%	QHJ85257.1
		L. crispatus phage UC164 [positions 6822 – 7259]	MAG: hypothetical protein [Bacteriophage sp.]	31.93%	QHJ77121.1
		L. crispatus phage UC164 [positions 17190 – 17600]	MAG: hypothetical protein [*Caudoviricetes* sp.]	62.77%	QHJ78071.1
	**Tail fiber protein**	*L. crispatus* phage UC093 [positions 20794 – 21219]	pyocin knob domain-containing protein [*Lactobacillus crispatus*]	97.14%	WP_232804795.1
		L. crispatus phage UC101	-	-	-
		L. crispatus phage UC119 [positions 22208 – 23296]	MAG TPA: tail-collar fiber protein [*Caudoviricetes* sp.]	82.66%	DAQ70487.1
		L. crispatus phage UC164 [positions 21498 – 23189]	MAG TPA: tail protein [*Caudoviricetes* sp.]	37.14%	DAZ21175.1
	**Tail protein**	*L. crispatus* phage UC093 [positions 6233 – 6619]	MAG: hypothetical protein [*Caudoviricetes* sp.]	36.97%	QHJ85637.1
		L. crispatus phage UC093 [positions 7404 – 7751]	MAG: hypothetical protein [*Caudoviricetes* sp.]	66.96%	QHJ84227.1
		L. crispatus phage UC093 [positions 15231 – 18944]	MAG TPA: tail protein [*Caudoviricetes* sp.]	67.04%	DAW18302.1

		L. crispatus phage UC101 [positions 15412 – 18738]	phage tail spike protein [*Lactobacillus crispatus*]	90.00%	WP_341025734.1
		L. crispatus phage UC101 [positions 21144 – 21758]	hypothetical protein [*Lactobacillus crispatus*]	100.00%	WP_005725835.1
		L. crispatus phage UC101 [positions 6394 – 6783]	MAG: hypothetical protein [*Caudoviricetes* sp.]	42.50%	QHJ85637.1
		L. crispatus phage UC101 [positions 7569 – 7913]	phage tail tube assembly chaperone [*Lactobacillus crispatus*]	95.58%	WP_061102297.1
		L. crispatus phage UC119 [positions 23313 – 25487]	MAG TPA: hypothetical protein [*Caudoviricetes* sp.]	55.38%	DAX91864.1
		L. crispatus phage UC119 [positions 43739 – 45415]	hypothetical protein HWC10_gp016 [*Lactobacillus* phage SAC12B]	52.63%	YP_009844171.1
		L. crispatus phage UC119 [positions 3672 – 4028]	MAG: hypothetical protein [Bacteriophage sp.]	61.90%	QHJ77576.1
		L. crispatus phage UC164 [positions 10479 – 10847]	MAG: hypothetical protein MSP0463_110 [Caudoviricetes sp.]	34.38%	XOS72567.1
	**Distal tail protein (Dit)**	*L. crispatus* phage UC093 [14461 – 15231]	phage tail protein [*Lactobacillus crispatus*]	98.83%	WP_060808450.1
		L. crispatus phage UC101 [14696 – 15412]	phage tail protein [*Lactobacillus crispatus*]	94.59%	WP_101883364.1
		L. crispatus phage UC119	-	-	-
		L. crispatus phage UC164	-	-	-
	**Tail length tape measure protein**	*L. crispatus* phage UC093 [positions 7971 – 14471]	tape measure protein [*Lactobacillus crispatus*]	99.91%	WP_060464083.1
		L. crispatus phage UC101 [positions 8131 – 14649]	tape measure protein [*Lactobacillus crispatus*]	94.27%	WP_061102298.1
		L. crispatus phage UC119 [positions 11234 – 16801]	Phage tape measure protein [*Lactobacillus crispatus*]	99.78%	WP_240407440.1
		L. crispatus phage UC164 [positions 3271 – 4068]	MAG: hypothetical protein [Bacteriophage sp.]	66.67%	QHJ78311.1
		L. crispatus phage UC164 [positions 11131 – 16206]	phage tail tape measure protein [*Lactobacillus crispatus*]	98.29%	WP_340858660.1
	**Tail sheath**	*L. crispatus* phage UC093	-	-	-
		L. crispatus phage UC101	-	-	-
		L. crispatus phage UC119 [positions 8776 – 10110]	MAG TPA: tail sheath protein [*Caudoviricetes* sp.]	48.07%	DAU04982.1
		L. crispatus phage UC164 [positions 8803 – 9972]	MAG TPA: tail sheath protein [*Caudoviricetes* sp.]	60.84%	DAU04982.1
	**Tail terminator**	*L. crispatus* phage UC093	-	-	-
		L. crispatus phage UC101	-	-	-

		L. crispatus phage UC119 [positions 8212 – 8772]	phage neck terminator protein [*Lactobacillus crispatus*]	99.45%	WP_240395076.1
		L. crispatus phage UC164 [positions 8258 – 8803]	phage neck terminator protein [*Lactobacillus crispatus*]	99.40%	WP_024129919.1
	**Tail completion or Neck1 protein**	*L. crispatus* phage UC093 [positions 5799 – 6236]	MAG: hypothetical protein [*Caudoviricetes* sp.]	58.39%	QHJ84224.1
		L. crispatus phage UC101 [positions 5957 – 6937]	MAG: hypothetical protein [*Caudoviricetes* sp.]	47.65%	QHJ84224.1
		L. crispatus phage UC119 [positions 7224 – 7820]	MAG: hypothetical protein [Bacteriophage sp.]	38.89%	QHJ77120.1
		L. crispatus phage UC164 [positions 7259 – 7855]	MAG: hypothetical protein [Bacteriophage sp.]	48.24%	QHJ77120.1
	**Major tail protein**	*L. crispatus* phage UC093 [positions 6601 – 7311]	phage tail protein [*Lactobacillus crispatus*]	100.00%	WP_060464085.1
		L. crispatus phage UC101 [positions 6765 – 7469]	phage tail protein [*Lactobacillus helveticus*]	91.45%	WP_046814301.1
		L. crispatus phage UC119	-	-	-
		L. crispatus phage UC164	-	-	-
	**Head-tail adaptor Ad1**	*L. crispatus* phage UC093	head-tail connector protein [*Lactobacillus crispatus*]	100.00%	WP_060464087.1
		L. crispatus phage UC101	head-tail connector protein [*Lactobacillus amylovorus*]	94.12%	WP_248616350.1
		L. crispatus phage UC119	-	-	-
		L. crispatus phage UC164	-	-	-
	**Head closure**	*L. crispatus* phage UC093	head-tail adaptor protein [*Lactobacillus crispatus*]	99.14%	WP_240396365.1
		L. crispatus phage UC101	phage major capsid protein [*Lactobacillus crispatus*]	98.62%	WP_133472241.1
		L. crispatus phage UC119	-	-	-
		L. crispatus phage UC164	MAG: hypothetical protein [Bacteriophage sp.]	39.02%	QHJ76526.1
	**Baseplate hub**	*L. crispatus* phage UC093	-	-	-
		L. crispatus phage UC101	-	-	-
		L. crispatus phage UC119 [positions 18195 – 19322]	MAG: hypothetical protein [Bacteriophage sp.]	52.12%	QHJ84844.1
		L. crispatus phage UC164 [positions 17600 – 18727]	MAG: hypothetical protein [Bacteriophage sp.]	52.41%	QHJ84844.1
	**Baseplate wedge**	*L. crispatus* phage UC093	-	-	-
		L. crispatus phage UC101	-	-	-
		L. crispatus phage UC119 [positions 19756 – 20163]	MAG TPA: Baseplate wedge protein [Myoviridae sp. ct1PY2]	99.25%	DAD82998.1
		L. crispatus phage UC119 [positions 20310 – 21341]	baseplate J/gp47 family protein [*Lactobacillus crispatus*]	94.17%	WP_060463459.1
		L. crispatus phage UC164 [positions 19158 – 19574]	MAG TPA: Baseplate wedge protein [Myoviridae sp. ct1PY2]	100.00%	DAD82998.1

		L. crispatus phage UC164 [positions 19564 – 20745]	baseplate J/gp47 family protein [*Lactobacillus crispatus*]	98.73%	WP_060463459.1
	**Baseplate spike**	*L. crispatus* phage UC093	-	-	-
		L. crispatus phage UC101	-	-	-
		L. crispatus phage UC119 [positions 19335 – 19808]	MAG TPA: baseplate protein [*Caudoviricetes* sp.]	51.92%	DAU04997.1
		L. crispatus phage UC164 [positions 18740 – 19213]	MAG TPA: baseplate protein [*Caudoviricetes* sp.]	53.21%	DAU04997.1
	**Structural protein**	*L. crispatus* phage UC093	-	-	-
		L. crispatus phage UC101	-	-	-
		L. crispatus phage UC119 [positions 21331 – 22179]	MAG TPA: Protein of unknown function (DUF2612) [*Caudoviricetes* sp.]	76.95%	DAX91866.1
		L. crispatus phage UC164 [positions 20735 – 21487]	MAG TPA: Protein of unknown function (DUF2612) [*Caudoviricetes* sp.]	74.47%	DAX91866.1
	**PblB-type receptor binding**	*L. crispatus* phage UC093 [positions 21220 – 21861]	MAG: protein of unknown function DUF4627 [Bacteriophage sp.]	87.94%	UVY21088.1
		L. crispatus phage UC101	-	-	-
		L. crispatus phage UC119	-	-	-
		L. crispatus phage UC164	-	-	-
**DNA replication**	**DNA helicase**	*L. crispatus* phage UC093 [positions 33682 – 35031]	helicase protein [*Lactobacillus* phage JNU_P5]	56.82%	QHJ74801.1
		L. crispatus phage UC101 [positions 32131 – 33474]	helicase protein [*Lactobacillus* phage JNU_P5]	54.59%	QHJ74801.1
		L. crispatus phage UC119	-	-	-
		L. crispatus phage UC164	-	-	-
	**DnaD-like helicase loader**	*L. crispatus* phage UC093	-	-	-
		L. crispatus phage UC101	-	-	-
		L. crispatus phage UC119 [positions 41282 – 42016]	MAG: DnaD [*Caudoviricetes* sp.]	51.70%	AXH73434.1
		L. crispatus phage UC164	-	-	-
	**DNA primase**	*L. crispatus* phage UC093 [positions 35759 – 38086]	DNA primase [*Lactobacillus* phage phiadh]	57.29%	NP_050131.1
		L. crispatus phage UC101 [positions 34059 – 36380]	DNA primase [*Lactobacillus* phage phiadh]	55.67%	NP_050131.1
		L. crispatus phage UC119	-	-	-
		L. crispatus phage UC164	-	-	-
	**Single strand DNA binding protein**	*L. crispatus* phage UC093 [positions 35196 – 35747]	hypothetical protein BN20_035 [*Lactobacillus* phage EV3]	31.79%	CUQ99458.1
		L. crispatus phage UC101 [positions 33477 – 34046]	single strand DNA binding protein [*Lactobacillus* phage phiadh]	34.64%	NP_050129.1

		L. crispatus phage UC119	-	-	-
		L. crispatus phage UC164	-	-	-
	**Sak4-like ssDNA annealing protein**	*L. crispatus* phage UC093 [positions 32693 – 33400]	NTP-binding motif protein [*Lactobacillus* phage P185]	51.97%	WFD53020.1
		L. crispatus phage UC101 [positions 31127 – 31849]	DNA-binding protein [*Streptococcus* phage CHPC1027]	44.29%	YP_010645118.1
		L. crispatus phage UC119	-	-	-
		L. crispatus phage UC164	-	-	-
	**Replication initiation protein**	*L. crispatus* phage UC093	-	-	-
		L. crispatus phage UC101	-	-	-
		L. crispatus phage UC119	-	-	-
		L. crispatus phage UC164 [positions 36907 – 37653]	conserved phage C-terminal domain-containing protein [*Limosilactobacillus mucosae*]	35.71%	WP_006500035.1
		L. crispatus phage UC093	-	-	-
	**DNA repair protein**	*L. crispatus* phage UC101	-	-	-
		L. crispatus phage UC119	-	-	-
		L. crispatus phage UC164 [positions 38152 – 38757]	MAG TPA: protein of unknown function DUF2466 [*Caudoviricetes* sp.]	97.44%	DAI45827.1
**Life Cycle Regulation**	**Integrase**	*L. crispatus* phage UC093 [positions 25843 – 27024]	recombinase [*Lactobacillus* phage mv4]	36.57%	AAC48859.1
		L. crispatus phage UC101 [positions 24614 – 25780]	integrase [*Caudoviricetes* sp.]	43.16%	CAI9750723.1
		L. crispatus phage UC119 [31842 – 32990]	MAG TPA: Integrase [*Caudoviricetes* sp.]	34.85%	DAW68993.1
		L. crispatus phage UC164 [positions 28249 – 29361]	integrase [*Caudoviricetes* sp.]	72.97%	CAI9750723.1
	**Transcriptional repressor**	*L. crispatus* phage UC093	-	-	-
		L. crispatus phage UC101 [positions 27926 – 28138]	MAG TPA: repressor protein [*Caudoviricetes* sp.]	44.93%	DAU85694.1
		L. crispatus phage UC119 [positions 35575 – 35793]	MAG TPA: Regulatory protein [*Caudoviricetes* sp.]	60.29%	DAT15792.1
		L. crispatus phage UC164 [positions 33945 – 34154]	MAG TPA: helix-turn-helix domain protein [*Caudoviricetes* sp.]	53.70%	DAU52925.1
		L. crispatus phage UC164 [positions 40824 – 41018]	MAG TPA: Regulatory protein [*Caudoviricetes* sp.]	100.00%	DAX91881.1
	**Transcriptional activator**	*L. crispatus* phage UC093 [positions 41382 – 41846]	ArpU family phage packaging/lysis transcriptional regulator [*Lactobacillus crispatus*]	97.99%	WP_230853088.1
		L. crispatus phage UC101 [positions 40504 – 40968]	ArpU family phage packaging/lysis transcriptional regulator [*Lactobacillus crispatus*]	98.66%	WP_230853088.1

		L. crispatus phage UC119 [positions 45887 – 46390]	MULTISPECIES: ArpU family phage packaging/lysis transcriptional regulator [Lactobacillaceae]	99.40%	WP_005724912.1
		L. crispatus phage UC164 [41009 – 41518]	hypothetical protein [*Lactobacillus crispatus*]	95.73%	WP_340850712.1
	**Transcriptional regulator**	*L. crispatus* phage UC093 [positions 28794 – 29138]	MAG TPA: repressor protein [*Caudoviricetes* sp.]	39.29%	DAM18963.1
		L. crispatus phage UC101 [positions 27324 – 27692]	MAG TPA: repressor protein [*Caudoviricetes* sp.]	40.00%	DAP38540.1
		L. crispatus phage UC119 [positions 34968 – 35336]	MAG: hypothetical protein [Cystoviridae sp.]	44.74%	QHJ85709.1
		L. crispatus phage UC164 [positions 30537 – 30878]	CI phage repressor protein [*Enterococcus* phage phiFL1C]	53.03%	ACZ63826.1
		L. crispatus phage UC164 [positions 37653 – 37931]	putative transcriptional regulator [*Lactobacillus* phage phiAQ113]	69.49%	YP_007173646.1
	**Superinfection exclusion**	*L. crispatus* phage UC093	-	-	-
		L. crispatus phage UC101 [positions 25937 – 26470]	hypothetical protein [*Lactobacillus crispatus*]	98.31%	WP_021355307.1
		L. crispatus phage UC119	-	-	-
		L. crispatus phage UC164 [positions 29421 – 30110]	hypothetical protein [*Lactobacillus crispatus*]	100.00%	WP_133476477.1
	**Recombinase**	*L. crispatus* phage UC093	-	-	-
		L. crispatus phage UC101	-	-	-
		L. crispatus phage UC119 [positions 40495 – 41280]	MAG TPA: hypothetical protein, partial [*Caudoviricetes* sp.]	72.00%	DAU06500.1
		L. crispatus phage UC164 [positions 35946 – 36905]	MAG: hypothetical protein [Bacteriophage sp.]	40.62%	QHJ76556.1
	**DNA methyltransferase**	*L. crispatus* phage UC093	-	-	-
		L. crispatus phage UC101	-	-	-
		L. crispatus phage UC119 [positions 39614 – 40492]	hypothetical protein CL1_31 [*Lactobacillus* phage CL1]	26.64%	YP_009206687.1
		L. crispatus phage UC164 [positions 35071 – 35946]	MAG TPA: Protein of unknown function (DUF1351) [*Caudoviricetes* sp.]	24.66%	DAM25014.1
	**Arc-like repressor**	*L. crispatus* phage UC093 [positions 41216 – 41335]	MAG TPA: Proline dehydrogenase/DNA Complex, Proline, Utilization, DNA, DNA [*Caudoviricetes* sp.]	48.65%	DAV67987.1
		L. crispatus phage UC093 [positions 43331 – 43468]	MAG: Arc-like DNA binding domain protein [Bacteriophage sp.]	45.45%	UVY61159.1
		L. crispatus phage UC101 [positions 40314 – 40457]	MAG TPA: Proline dehydrogenase/DNA Complex, Proline, Utilization, DNA, DNA [*Caudoviricetes* sp.]	48.65%	DAV67987.1
		L. crispatus phage UC119	-	-	-
		L. crispatus phage UC164	-	-	-

	**Endonuclease**	*L. crispatus* phage UC093 [positions 40863 – 41201]	MAG TPA: Nuclease [*Caudoviricetes* sp.]	99.11%	DAR79682.1
		L. crispatus phage UC101 [positions 39472 – 39810]	MAG TPA: Nuclease [*Caudoviricetes* sp.]	76.79%	DAR79682.1
		L. crispatus phage UC119	-	-	-
		L. crispatus phage UC164 [positions 40145 – 40525]	MAG TPA: Nuclease [*Caudoviricetes* sp.]	46.30%	DAT42427.1
	**HNH Endonuclease**	*L. crispatus* phage UC093 [positions 32125 – 32700]	MAG TPA: homing endonuclease [*Caudoviricetes* sp.]	44.32%	DAX40189.1
		L. crispatus phage UC093 [positions 42117 – 42710]	MAG: HNH endonuclease [Straboviridae sp.]	94.85%	XQU59695.1
		L. crispatus phage UC093 [positions 43555 – 44406]	MAG: HNH endonuclease [*Caudoviricetes* sp.]	35.21%	XOS49136.1
		L. crispatus phage UC101 [positions 41241 – 41831]	MAG: HNH endonuclease [Straboviridae sp.]	100.00%	XQU59695.1
		L. crispatus phage UC119	-	-	-
		L. crispatus phage UC164	-	-	-
	**ParB-like partition nuclease**	*L. crispatus* phage UC093	-	-	-
		L. crispatus phage UC101	-	-	-
		L. crispatus phage UC119	-	-	-
		L. crispatus phage UC164 [positions 43212 – 43385]	MAG TPA: ParB protein [*Caudoviricetes* sp.]	84.21%	DAJ54861.1
	**Anti-repressor Ant**	*L. crispatus* phage UC093 [positions 30019 – 30768]	antirepressor [*Lactobacillus* phage vB_Lga_AB1]	74.49%	QRD99507.1
		L. crispatus phage UC101 [positions 28139 – 28936]	MAG TPA: hypothetical protein [*Caudoviricetes* sp.]	50.96%	DAU85720.1
		L. crispatus phage UC119 [positions 36175 – 36993]	antirepressor protein [*Lactobacillus* phage Ldl1]	64.66%	YP_009126518.1
		L. crispatus phage UC164 [positions 39283 – 39972]	antirepressor [*Lactobacillus* phage CR191]	62.72%	WFD52874.1
	**Plasmid antitoxin with HTH domain**	*L. crispatus* phage UC093	-	-	-
		L. crispatus phage UC101	-	-	-
		L. crispatus phage UC119	-	-	-
		L. crispatus phage UC164 [positions 32799 – 33029]	MAG TPA: Helix-turn-helix XRE-family like protein [*Caudoviricetes* sp.]	50.72%	DAR77088.1
	**Metallo-protease**	*L. crispatus* phage UC093 [positions 28387 – 28791]	ImmA/IrrE family metallo-endopeptidase [*Lactobacillus* phage CV244]	47.01%	WFD52927.1
		L. crispatus phage UC101 [positions 26519 – 27322]	MAG TPA: IrrE protein [*Caudoviricetes* sp.]	30.04%	DAE74143.1

		L. crispatus phage UC119 [positions 34507 – 34959]	ImmA/IrrE family metallo-endopeptidase [*Lactobacillus* phage CV244]	62.39%	WFD52927.1
		L. crispatus phage UC164 [positions 30121 – 30525]	MAG TPA: IrrE protein [Caudoviricetes sp.]	75.58%	DAX91846.1
**Lysis**	**Endolysin**	*L. crispatus* phage UC093 [positions 24039 – 24962]	MAG: glycosyl hydrolase family 25 [Bacteriophage sp.]	71.01%	UWG70141.1
		L. crispatus phage UC101 [positions 23473 – 24318]	endolysin [*Lactobacillus* phage KC5a]	62.14%	YP_529896.1
		L. crispatus phage UC119 [positions 16813 – 17772]	MAG: hypothetical protein [Bacteriophage sp.]	53.08%	QHJ77112.1
		L. crispatus phage UC119 [positions 29214 – 30335]	endolysin [*Lactobacillus* phage phiAQ113]	83.91%	YP_007173630.1
		L. crispatus phage UC164 [positions 16218 – 17177]	MAG: hypothetical protein [Bacteriophage sp.]	53.08%	QHJ77112.1
		L. crispatus phage UC164 [positions 26550 – 27671]	endolysin [*Lactobacillus* phage phiAQ113]	84.72%	YP_007173630.1
	**Excisionase and transcriptional regulator**	*L. crispatus* phage UC093 [positions 30771 – 30989]	MAG TPA: helix-turn-helix domain protein [Bacteriophage sp.]	54.93%	DAG85243.1
		L. crispatus phage UC101 [positions 29773 – 29988]	MAG TPA: helix-turn-helix domain protein [Bacteriophage sp.]	54.93%	DAG85243.1
		L. crispatus phage UC119	-	-	-
		L. crispatus phage UC164	-	-	-
	**Holin**	*L. crispatus* phage UC093 [positions 22833 – 23387]	MAG TPA: hypothetical protein [*Caudoviricetes* sp.]	99.46%	DAX38038.1
		L. crispatus phage UC093 [position 23594 – 24049]	phage holin [*Lactobacillus crispatus*]	92.72%	WP_151495269.1
		L. crispatus phage UC101 [positions 22436 – 22852]	holin [*Lactobacillus* phage phiadh]	33.33%	NP_050167.1
		L. crispatus phage UC101 [positions 23085 – 23480]	phage holin [*Lactobacillus crispatus*]	97.71%	WP_060463584.1
		L. crispatus phage UC119 [positions 27875 – 28276]	holin [*Lactobacillus* phage CR191]	68.61%	WFD52863.1
		L. crispatus phage UC119 [positions 28744 – 29142]	phage holin, LLH family [*Lactobacillus crispatus*]	70.45%	WP_285025324.1
		L. crispatus phage UC164 [positions 23208 - 23600]	phage holin, LLH family [*Lactobacillus crispatus*]	96.97%	WP_285025324.1
		L. crispatus phage UC164 [positions 26053 – 26451]	MAG TPA: hypothetical protein [*Caudoviricetes* sp.]	30.48%	DAS87140.1

Supplementary Table S3. Alignment of induced phage genomes to predicted prophage regions in corresponding *Lactobacillus crispatus* host strains

Host Strain ID	Predicted phage contig ID	Tools calling region	CheckV Quality	PHASTER prediction	Prophage contig length (bp)	Induced Phage ID	Induced Phage Length (bp)	% Pairwise Identity with Induced Phage
UC0930205	NODE4	VirSorter2, CheckV, PHASTER	Complete	Intact	42,405	UC093	44,507	100.00%
	NODE17	VirSorter2, CheckV, PHASTER	Medium quality	Incomplete	31,532	UC093	44,507	34.20%
	NODE19	VirSorter2, CheckV, PHASTER	Medium quality	Incomplete	30,580	UC093	44,507	33.30%
UC1010110	NODE9	VirSorter2, CheckV, PHASTER	Complete	Intact	42,955	UC101	42,893	100.00%
	NODE4	VirSorter2, CheckV, PHASTER	High quality	Incomplete	51,736	UC101	42,893	37.10%
	NODE2	VirSorter2, CheckV, PHASTER	Medium quality	Incomplete	42,308	UC101	42,893	38.60%
	NODE32	VirSorter2, CheckV	Medium quality	-	23,956	UC101	42,893	29.70%
UC1190127	NODE1	VirSorter2, CheckV, PHASTER	High quality	Intact	38,203	UC119	48,283	39.60%
UC1640140	NODE7	VirSorter2, CheckV, PHASTER	Medium quality	Incomplete	39,584	UC164	44,440	100.00%
